# Climate dependent feasibility of closing water and nutrient cycles in industrial aqua-agriculture

**DOI:** 10.1016/j.isci.2026.116266

**Published:** 2026-06-08

**Authors:** Milan de Korte, Joris Bergman, L. Gerard van Willigenburg, Victor Lobanov, Alyssa Joyce, Xiaodong Cheng, Karel J. Keesman

**Affiliations:** 1 Mathematical and Statistical Methods (Biometris), Wageningen University and Research, P.O. Box 16, 6700 AA, Wageningen, the Netherlands; 2 Wetsus, European Centre of Excellence for Sustainable Water Technology, Leeuwarden 8911 AD, the Netherlands; 3 Department of Marine Science, Gothenburg University, Box 461, Göteborg 40530, Sweden

**Keywords:** Farming management, Agricultural engineering, Relation between agriculture and environment, Aquaculture

## Abstract

In integrated aqua-agriculture, matching waste-derived nutrient supply with crop demand while maintaining optimal fish and crop conditions remains challenging. We formulate a closed-loop aquaculture-hydroponics network with anaerobic digestion as a constrained optimal-control problem and combine static sizing with climate-driven dynamic flow optimization for contrasting climates. Under weak seasonality (Jakarta), internal wastes (aquaculture water, fish sludge, and plant residues) support discharge-free operation with water, nitrogen, and phosphorus use-efficiencies of 100%, 79%, and 72%. As seasonality increases (Cairo, Amsterdam), feasibility and robustness decline due to shifting demand and limited phosphorus recoverability. Using manure as co-digestion substrate restores closed-cycle operation without synthetic fertilizers. The optimizer prefers pig manure in Cairo and chicken manure in Amsterdam, reflecting climate-specific N:P deficits. Relative to climate-matched decoupled aquaponics, optimized integrated networks cut water, nitrogen, and phosphorus waste by 86%, 46%, and 35%. Overall, the framework enables climate-robust design of circular aqua-agriculture networks, reducing resource use and pollution.

## Highlights

• Integrating anaerobic digestion enables circular industrial aqua-agriculture
• Internal waste allows discharge- and fertilizer-free operation in tropical climates
• Increasing seasonality reduces both water and phosphorus loop closure
• Co-digestion restores loop closure with climate-dependent optimal manure type

## Graphical abstract

**Figure:**
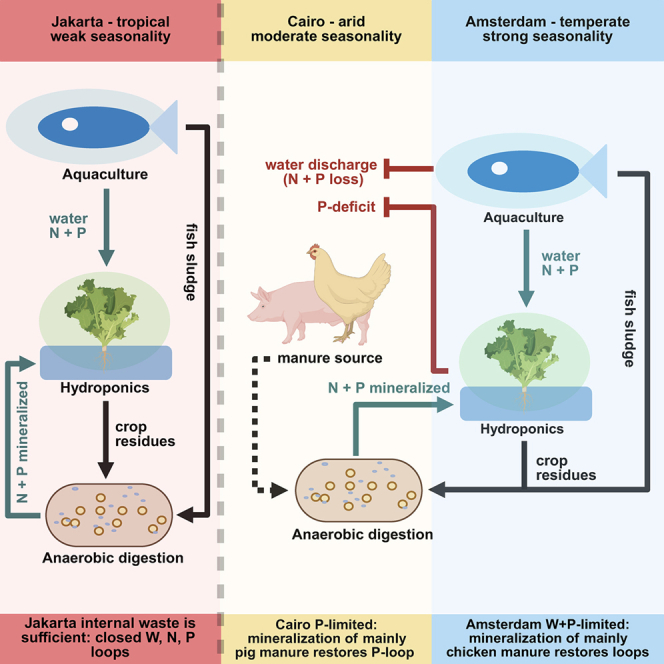

## Introduction

Over the last few decades, global food production has expanded rapidly, placing increasing pressure on natural resources. Fresh water, arable land, and essential mineral nutrients such as nitrogen and phosphorus are already being used beyond safe planetary limits, threatening ecosystem integrity, the sustainable availability of renewable freshwater resources, and finite phosphate-rock reserves.^1^ Improving self-sufficiency and use-efficiency by replacing virgin inputs with resources recovered from waste streams is one way to more sustainably meet future food demand.^2^ In this context, integrated aqua-agriculture offers a promising pathway toward closed-resource-cycle food production by growing crops using the metabolic waste products of fish or other aquatic animals as nutrient sources.^3^

Aquaculture is the fastest-growing segment of the global food industry and has recently surpassed capture fisheries in seafood production for human consumption.^4^ This shift can be attributed to the limitations and decline of wild fisheries stocks, as well as technological advancements that have enhanced aquaculture productivity.^5^ As the industry grows, an increasing amount of nutrient-rich waste is produced, particularly nitrogen and phosphorus. Fish retain only about half of the nitrogen and phosphorus supplied in feed, with the remainder released to the culture water as dissolved and particulate waste through gill excretion of ammonia, urine and feces, and feed leftovers.^6^^,^^7^ Nitrogen-containing compounds, such as proteins, are catabolized, releasing toxic ammonia, which in recirculating aquaculture systems (RAS) is controlled by biological nitrification in biofilters. However, once nitrate concentrations surpass tolerable limits, part of the water is often discharged without sufficient treatment.^8^

Integrating RAS with protected hydroponic systems (i.e., soil-less crop production) can substantially improve water and nitrogen use-efficiency by reusing nutrient-rich aquaculture water for crop production.^9^ In contrast to nitrogen, aquaculture water typically contains only small amounts of dissolved phosphorus because inorganic phosphates excreted by fish bind to feed particles and fecal matter, which are removed through mechanical filtration as concentrated sludge.^10^ Anaerobic digesters can convert this sludge into an organic fertilizer containing plant-available mineral forms such as phosphates and ammonium.^11^ In addition to fish sludge, anaerobic digestion can treat and recycle nitrogen and phosphorus from other organic farm wastes, thereby contributing to the nitrogen and phosphorus recovery that is possible within an industrial aqua-agriculture network.^12^^,^^13^^,^^14^ Integrating anaerobic digestion can simultaneously prevent aquaculture from releasing nutrients into the environment and reduce reliance on synthetic fertilizers for plant production.

While anaerobic digestion offers many advantages, its integration also increases the level of complexity. A key challenge is to identify system dimensions and corresponding flows that match waste-derived nutrient supply to crop nutrient demand without synthetic fertilizers or allowing nutrients to vary between tolerable levels in both fish or crops components.^15^ This is particularly challenging in regions with seasonal climates, given that crop nutrient uptake is largely driven by the climate-dependent crop transpiration rate.^16^ As transpiration rates drop from summer to winter, a larger plant cultivation area is needed to balance nitrogen and phosphorus inputs from aquaculture and subsequent sludge digestion. A recent optimization study demonstrated that this supply-demand mismatch in integrated aqua-agriculture can be managed by adopting a staggered production strategy and sizing the protected cultivation area to meet winter requirements, supplemented by an external mineralizable organic source.^17^ For example, manure can be diluted to mitigate ammonia toxicity^18^ and co-digested to boost nutrient availability during periods of high demand, while staggered fish and crop production helps smooth feed inputs and crop transpiration rates. However, this specific strategy has so far only been investigated in steady-state scenarios in the Netherlands and remains untested under dynamic conditions across contrasting climates.

The aim of this model-based study was to quantify under what climatic conditions industrial aqua-agriculture networks can operate in closed water and nutrient cycles, i.e., reusing aquaculture water for irrigation without synthetic fertilizer addition, under realistic daily weather variability, and how this feasibility changes with increasing seasonality. We represent the network with coupled sub-models for water and nutrient dynamics in a RAS producing Nile Tilapia, a hydroponic production system (HPS) growing lettuce, and two anaerobic digesters that separately treat fish sludge and crop residues. Using these models, we applied combined static and dynamic optimization to determine location-specific optimal designs and time-varying flows that minimize discharge and synthetic fertilizer inputs while maintaining fish- and crop water-quality constraints.

We evaluated the framework for three contrasting climates using historical weather data. We started with Jakarta, Indonesia, which has a relatively stable tropical climate. The Indonesian aquaculture sector has expanded rapidly in recent decades, and Jakarta is one of the most populous and climate-vulnerable cities globally.^19^ We then considered Cairo, Egypt, with a two-seasonal, arid climate characterized by very hot summers and mild winters. Egypt is the largest aquaculture producer in Africa but already relies on water beyond its internal renewable sources, making it increasingly prone to future water stress.^20^ Finally, to explore the potential of sustainable integrated aqua-agriculture in strong temperate climates, we selected the Netherlands, where the highly variable four-season climate in Amsterdam makes it the most challenging of the three case studies. For each location, we first assessed a baseline configuration relying only on internal waste streams (aquaculture water, fish sludge, and plant residues). Where internal recycling proved constrained, we analyzed an extended configuration with manure co-digestion and quantified when it restores operational flexibility, including which manure type (chicken, pig, or cattle) is preferred under different seasonal demand profiles. We also benchmarked each location’s best-performing integrated configuration against an optimized decoupled aquaponics (DAP) reference network (RAS-HPS without anaerobic digestion) under identical system boundaries. To the best of our knowledge, no model study has been conducted that explores closure of nutrient and water cycles in integrated aqua-agriculture networks across different climates under dynamic conditions.

**Figure 1:**
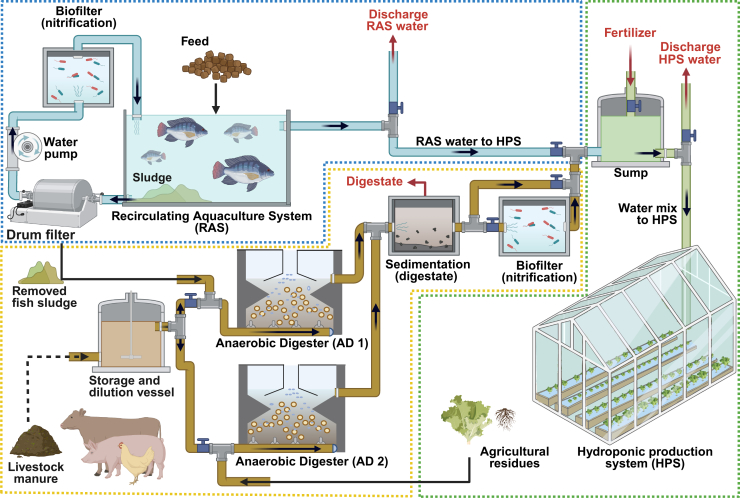
Simplified material flow diagram of the industrial aqua-agriculture network The blue dashed box represents the recirculating aquaculture system (RAS), the green dashed box the hydroponic production system (HPS), the yellow dashed box the anaerobic digestion system (ADS). The blue valves depict the location of the decision variables used in the model and the red arrows represent the streams that reduce the network performance in terms of water and nutrient use-efficiency or nutrient self-sufficiency. The decoupled aquaponics network only consists of the RAS and HPS subsystem whereas the addition of livestock manure (black dashed line) is only used in the extended integrated aqua-agriculture network. Created in BioRender. Korte, M. (2026) *https://BioRender.com/scyfszq*.

Figure 1 shows an overview of each submodel used to describe the different set-ups explored in this study. Analyzing these set-ups with a year-scale optimal-control formulation, we show that the internal-waste baseline can maintain fish- and crop water-quality constraints under weak seasonality (Jakarta), becomes marginally phosphorus-limited under moderate seasonality (Cairo), and under strong seasonality (Amsterdam) requires discharge and/or external supplementation to remain feasible. A ±20% parameter perturbation analysis further shows that performance as measured by the key performance indicators (water/nutrient use-efficiency and nutrient self-sufficiency) is largely insensitive to parameter perturbations in Jakarta, whereas in Cairo and Amsterdam manure co-digestion restores N and P balance and increases robustness by providing an additional lever over the N:P supply ratio. Finally, benchmarking against optimized decoupled aquaponics confirms that integrating anaerobic digestion reduces water and nutrient losses and lowers virgin input requirements.

## Results

### Computational performance

The following simulation results were obtained using the IPOPT solver and reached the prescribed non-linear problem optimality error tolerance below 10^−5^ across all scenarios with an average computation time of 183 s per annual simulation on a laptop with an Intel Core i5-1235U CPU and 16 GB RAM (Windows 11, Python v3.11.13, GEKKO v1.3.0, IPOPT v3.12, linear solver: MA57). This indicates that the framework is computationally efficient and hence suitable for real-time implementation.

### Weak seasonality allows closed-cycle operation on internal waste streams

**Figure 2:**
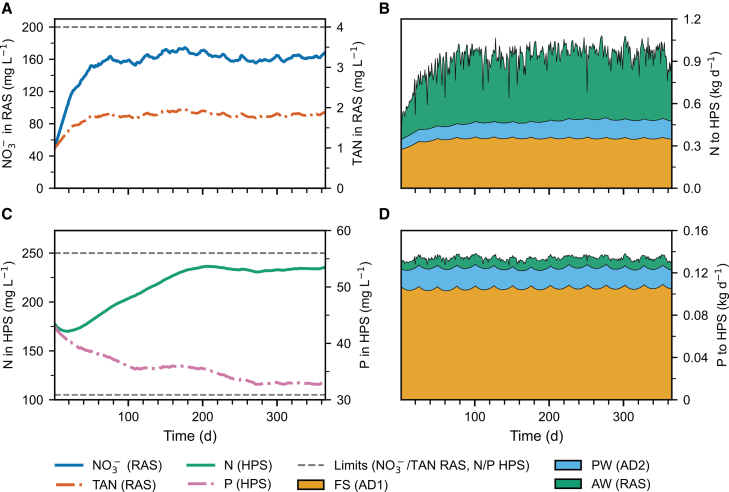
Nutrient and mass transfer profiles of the Jakarta case study (A) Shows the nitrate (NO_3_^−^) and total ammonia nitrogen (TAN) concentration for a 100 m^3^ recirculating aquaculture system (RAS). (B) Displays the N contributions of mineralized plant waste (PW), fish sludge (FS), and aquaculture-water transfer (AW) to the HPS. (C) Shows the total nitrogen (N) and phosphorus (P) profiles for the hydroponic production system (HPS) with a 1530 m^2^ cultivation area. (D) Shows the P contributions of mineralized plant waste (PW), fish sludge (FS), and aquaculture-water transfer (AW) to the HPS.

Under Jakarta’s relatively stable tropical climate (temperature, humidity, and radiation are similar year-round), the optimal control solution maintained nitrate and total ammonia nitrogen in the RAS below toxic thresholds while simultaneously keeping the hydroponic nutrient solution within the prescribed crop ranges (Figures 2A–2C). The greenhouse was therefore operated exclusively using nutrients from internal waste streams and without any water discharge to the environment, which resulted in 100% nitrogen and phosphorus self-sufficiency (NSS, PSS), 100% water use-efficiency (WUE), and a nitrogen and phosphorus use-efficiency (NUE, PUE) of 79% and 72%.

**Figure 3:**
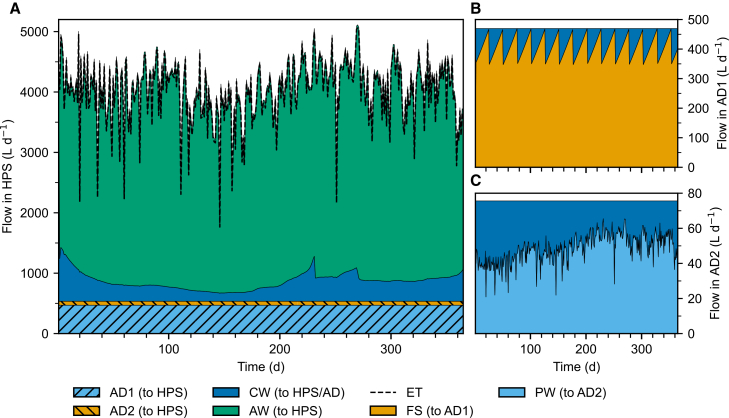
Hydraulic flow profiles of the Jakarta case study (A) Shows the water flows received by the hydroponic production system (HPS) from two anaerobic digesters, one digesting fish sludge (AD1) and another digesting plant waste (AD2), the recirculating aquaculture system (AW), and an external clean water source (CW). Together, they match the outgoing HPS evapotranspiration flux (ET) in Jakarta (indicated by the black dashed line). (B and C) Show the digester inflows from fish sludge (FS), plant waste (PW), and additional clean water to keep reactor volumes constant (CW1, CW2). The reactor inflows are determined based on a predefined range of total solid and volatile solid loading rates.

At the optimal design, the 1530 m^2^ HPS (306 m^3^ irrigation volume) received most of its nitrogen (54%) and a smaller share of its P (7%) directly via aquaculture water transfers (Figures 2B–2D). Phosphorus largely entered the hydroponic loop through mineralization of captured solids. A 14 m^3^ digester treating fish sludge (30-day HRT) supplied 32% of the hydroponic N and 79% of its P. A second, smaller digester (1.7 m^3^, 25-day HRT) mineralized inedible lettuce residues and provided the remaining nutrient fractions. The flows used to balance nutrient supply and demand are illustrated in Figure 3.

Hydroponic volume only decreased in response to transpiration. Transpiration losses were offset by steady digester effluents, supplemented by aquaculture water and clean water to satisfy volume constraints (Figure 3A). Variations in fish-sludge inflow reflected the staggered fish production strategy, while the addition of water ensured constant reactor volumes during periods of low fish sludge production (Figure 3B). In the digester handling inedible lettuce residues, clean water was likewise added to keep the reactor volume constant as lettuce-waste production fluctuated due to weather conditions and changes in hydroponic nutrient concentrations (Figure 3C). Despite variation in fish feed and lettuce waste supply, digester feed concentrations and volatile-solids loading rates in both reactors remained within, or close to, the prescribed feasibility bounds throughout the year.

### Seasonality narrows the feasible operating window and increases sensitivity

Compared to Cairo and Amsterdam (results shown in Figures S1 and S2), Jakarta represents the most favorable case, where relatively constant evapotranspiration keeps crop uptake easily aligned with the waste-derived nutrient supply. In more seasonal climates, the optimizer must instead navigate through alternating regimes where high-demand periods risk nutrient deficits and low-demand periods promote accumulation. In Cairo, the optimizer could still manage this trade-off without liquid discharge at an optimal HPS area of 1350 m^2^, maintaining full irrigation closure (WUE = 100%, NUE = 79%, PUE = 72%) and near-complete phosphorus self-sufficiency (PSS ≈ 99%). In Amsterdam, stronger seasonality increased the mismatch between demand and waste-derived supply, making periodic discharge and external inputs unavoidable at the optimal HPS area of 2700 m^2^ and lowering WUE, NUE, PUE, and PSS to 93%, 75%, 70%, and 82%, respectively.

**Figure 4:**
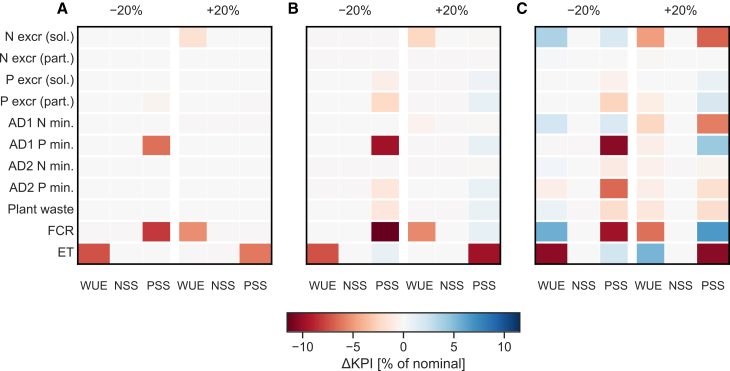
Sensitivity of the key performance indicators to ±20% perturbations in key parameters (A) Jakarta (1530 m^2^; WUE = 100%, NSS = 100%, PSS = 100%). (B) Cairo (1350 m^2^; WUE = 100%, NSS = 100%, PSS = 99%). (C) Amsterdam (2700 m^2^; WUE = 93%, NSS = 100%, PSS = 82%). Each image shows the relative change in water use efficiency (WUE), nitrogen self-sufficiency (NSS), and phosphorus self-sufficiency (PSS) compared with the corresponding nominal optimization, expressed as ΔKPI (% of nominal). Perturbed parameters are, fish N and P excretion split between soluble and particulate forms, N and P mineralization coefficients in AD1 and AD2, plant-waste generation, feed conversion ratio (FCR), and crop evapotranspiration (ET). Columns group the −20% and +20% perturbations for each KPI. Colors indicate improvement (blue) or deterioration (red) relative to the nominal case, with white denoting zero change.

Together, the Cairo and Amsterdam optima indicate that as seasonal variability increases, the feasible operating window narrows and performance becomes more sensitive to biological and operational uncertainty. To quantify this sensitivity, we re-solved the optimal control problem under ±20% perturbations of key parameters (Figure 4). Under perturbations, feasibility is still enforced through the path and balance constraints, but maintaining those constraints may require additional discharge and/or external nutrient inputs. We therefore use WUE, NSS, and PSS as sensitivity indicators that measure how strongly closed-loop performance decreases under uncertainty.

Across all climates, the sensitivity patterns are dominated by evapotranspiration (ET) and feed conversion ratio (FCR), which shift crop nutrient demand and waste-derived nutrient loading. The largest WUE reductions under ±20% perturbations were 7.3% in Jakarta and Cairo (both driven by −20% ET) and 9.6% in Amsterdam (−20% ET). The largest PSS reductions were 8.2% in Jakarta (−20% FCR), 11.4% in Cairo (−20% FCR), and 5.4% in Amsterdam (+20% FCR). In contrast, excretion-partitioning parameters and most mineralization coefficients caused only minor KPI changes within ±20%. Among the digestion-related parameters, the largest effect was a 20% reduction in the AD1 P mineralization coefficient, which reduced PSS by 9.6% in Amsterdam, 8.6% in Cairo, and 6.3% in Jakarta. NSS remains robust in all cases, whereas the higher sensitivity in PSS confirms that P recoverability becomes the critical bottleneck under uncertainty.

Jakarta shows the weakest response, consistent with its stable ET regime. KPI reduction occurs mainly when ET decreases, which reduces crop uptake and triggers discharge (lower WUE), and when effective phosphorus recovery decreases (e.g., lower AD1 P mineralization or lower waste generation), which reduces PSS.

Cairo exhibits the same mechanisms as Jakarta but with a stronger PSS response. Reduced ET lowers crop demand, increasing the risk of accumulation and triggering discharge (WUE decreases), whereas increased ET exposes phosphorus limitation during peak demand, requiring external P inputs and reducing PSS. Taken together, this indicates that in two-season hot climates, peak-demand periods can amplify P limitation, particularly when biological P recovery is simultaneously reduced. Minor improvements in PSS are measured as well for positive perturbations that cause an increase in P supply.

Amsterdam is the most sensitive overall. Strong seasonality pushes the network into both oversupply (cold, low-ET periods) and undersupply (warm, high-ET periods), so ±20% perturbations can have comparable but opposite effects. In this regime, perturbations that increase net N loading (e.g., higher fish excretion, higher mineralization, or higher plant-waste availability) can worsen performance by accelerating nitrate accumulation, increasing the need for discharge and thereby reducing WUE (and, indirectly, PSS). Conversely, lower ET further increases accumulation pressure and reduces WUE, whereas higher ET raises crop demand and shifts the limiting factor toward phosphorus availability, reducing PSS. Overall, FCR and AD1 P mineralization remain the strongest drivers of PSS.

### Manure co-digestion restores closed-loop feasibility and robustness in seasonal climates

The results and sensitivity analysis from the base configurations indicate that closed-loop operation on internal waste streams is robust under tropical conditions, but becomes increasingly constrained as seasonality intensifies. While NSS remains stable across climates, PSS decreases under a wide range of parameter perturbations, especially in Amsterdam and, to a lesser extent, in Cairo, reflecting phosphorus limitation during high-demand periods and nitrogen accumulation beyond the target range during low-demand periods. We therefore evaluated an extended configuration in which manure is co-digested to provide an additional mineralizable nutrient source and increase operational flexibility. This extension was analyzed for Cairo and Amsterdam, where it is most relevant.

**Figure 5:**
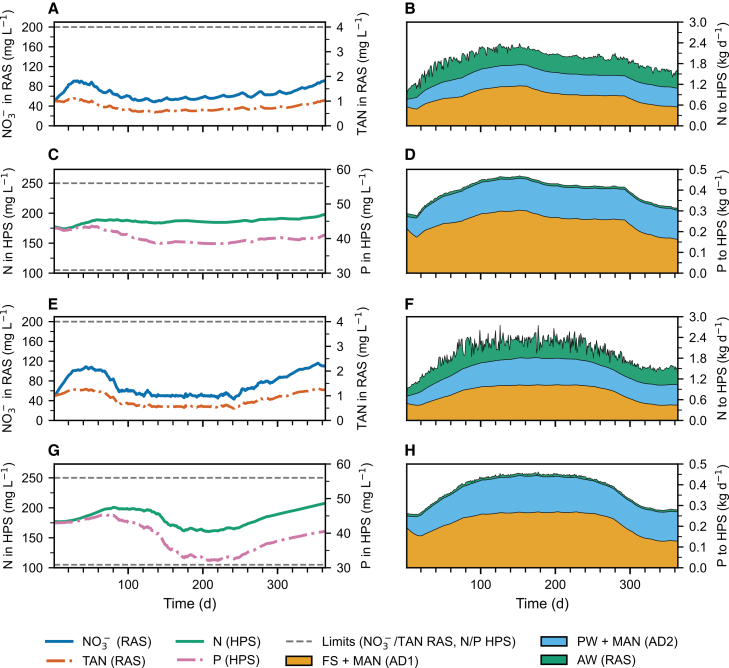
Nutrient and mass transfer profiles of the Cairo (AB-EF) and Amsterdam (CD-GH) case studies (A–E) Show the nitrate (NO_3_^−^) and total ammonia nitrogen (TAN) concentrations for a 100 m^3^ recirculating aquaculture system (RAS) in Cairo and Amsterdam. (B–F) Show the amount of N received by the HPS from fish sludge (FS + MAN) and plant waste, both co-digested with a manure mixture (PW + MAN), and aquaculture-water transfer (AW). (C and G) Show the total nitrogen (N) and phosphorus (P) profiles for the hydroponic production system (HPS) with a 3220 m^2^ and 5130 m^2^ cultivation area for Cairo and Amsterdam. (D–H) Show the amount of P received by the HPS from fish sludge (FS + MAN) and plant waste, both co-digested with a manure mixture (PW + MAN), and aquaculture-water transfer (AW).

For both locations, the extended configuration enabled fully closed-cycle operation of the irrigation and nutrient supply, thereby achieving 100% performance in WUE, NSS, and PSS. Overall nutrient-use efficiencies achieved for Cairo: NUE = 76%, PUE = 59%, and in Amsterdam: NUE = 75%, PUE = 60%, reflecting nutrient losses through non-recovered digestate and denitrification streams within the network boundaries. Figure 5 presents the nutrient profiles and source contributions in the manure-extended configuration. In both cases, RAS water-quality indicators (NO_3_^−^, TAN) remain well below thresholds throughout the year, and HPS nutrient concentrations remain within their target bands. In Cairo, the dominant share of N and P is supplied by pig manure (44.5% and 47.7%), followed by fish sludge (25.6% and 28.0%), plant waste (17.8% and 13.5%), and chicken manure (12.0% and 10.8%). In Amsterdam, chicken manure becomes dominant (44.7% and 43.6%), followed by fish sludge (26.3% and 29.8%), plant waste (17.1% and 13.6%), and pig manure (11.8% and 12.6%). Cow manure remained unused in both locations, which demonstrates that the optimizer selects co-substrates that best match the location-specific N:P requirement under seasonal demand.

**Figure 6:**
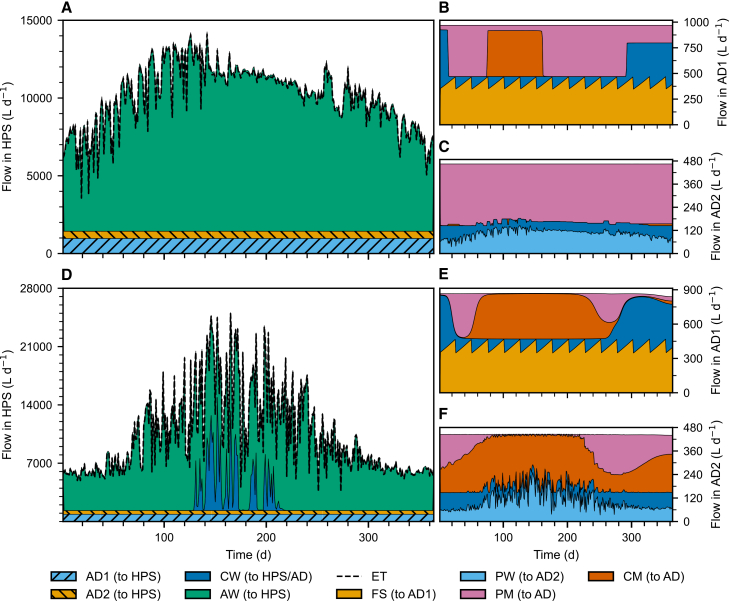
Hydraulic flow profiles of the Cairo (ABC) and Amsterdam (DEF) case studies (A–D) Shows the amount of water the HPS receives from two anaerobic digesters, one co-digesting fish sludge with a manure mixture (AD1) and the other co-digesting plant waste with a manure mixture (AD2), an external fresh water source (CW), and the recirculating aquaculture system (AW). These flows align with the greenhouse evapotranspiration (ET) denoted by the black dashed line. (B–F) Show the incoming anaerobic digester flows from fish sludge (FS), plant waste (PW), chicken manure (CM), pig manure (PM). Reactor inflows are determined based on a predefined range of total solid and volatile solid loading rates.

**Figure 7:**
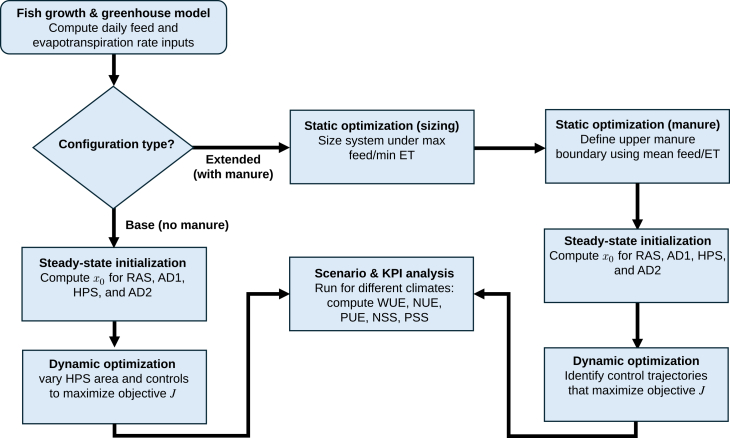
Flow chart of the modeling and optimization procedure

Figure 6 shows the hydraulic profiles of the manure-extended networks. Seasonal greenhouse evapotranspiration drives the net irrigation demand (Figures 6A–6D), which is met by a combination of aquaculture-water transfer, clean-water, and effluent from AD1-AD2. The digester inflows (Figures 6B–6F) show how the optimizer modulates manure inflows to adjust N:P ratios and adjusts volume based on the season. In Cairo pig manure represents 85% of the total manure volumetric flow input whereas in Amsterdam 71% of the flow originates from chicken manure. The required reactor volumes were 29 m^3^ and 26 m^3^ for AD1 and 12 m^3^ and 11 m^3^ for AD2 in Cairo and Amsterdam, respectively. Larger reactor volumes are required compared to the base-configuration to accommodate the additional manure flows that are needed to feed the oversized HPS area. For both locations, volatile solids (VS) loading rates and total solids (TS) levels were kept very close in range of the optimum. Finally, repeating the ±20% perturbation analysis for the manure-extended configurations showed that co-digestion completely dampened sensitivity. None of the perturbations caused changes in the KPIs (results not shown). The overall workflow used to obtain these base and manure-extended network configurations, including climate-data preprocessing, static sizing, dynamic optimization, and KPI evaluation, is summarized in Figure 7.

### Integrated anaerobic digestion outperforms optimized decoupled aquaponics

We evaluated each optimized network using water, nitrogen, and phosphorus use efficiencies (WUE, NUE, and PUE), defined as one minus the ratio of annual losses to annual inputs. We additionally report nitrogen and PSS (NSS, PSS), indicating the fraction of hydroponic nutrient demand met from internal sources (fish and plant waste, and manure optionally). For WUE, the wasted water stream excludes water contained in unutilized wet-solid material waste streams (digestate, sludge, and plant waste), which is reported separately, because it represents only a small fraction of total water inputs. Table 1 compares the indicators and associated resource flows with optimized decoupled aquaponics (DAP) reference network without anaerobic digestion under identical climate conditions. The decision variables and constraints underlying these optimizations are summarized in Table 2, and the KPI definitions used to calculate the indicators in Table 1 are provided in Table 3. DAP nutrient and fertilizer supplementation profiles are shown in Figures S5–S7.

**Table 1:** Optimized industrial aqua-agriculture (IAA) networks compared with optimized decoupled aquaponics (DAP)

Network: HPS area (m^2^): Resource	B-IAA Jak. 1530	DAP Jak. 1800	E-IAA Cai. 3220	DAP Cai. 1575	E-IAA Ams. 5130	DAP Ams. 2250
WUE (%)	100	100	100	100	100	100
NUE (%)	79	58	76	64	75	63
PUE (%)	72	53	59	60	60	58
NSS (%)	100	100	100	100	100	100
PSS (%)	100	8	100	7	100	8
Yield lettuce (kg m^−2^ y^−1^)	42	27	47	30	31	28
Water input (L m^−2^ y^−1^)	955	1237	1140	1232	730	1284
Water loss via wet solids^a^ (L m^−2^ y^−1^)	13	89	16	94	9	94
N Input (gN m^−2^ y^−1^)	437	372	335	425	214	298
N wasted (gN m^−2^ y^−1^)	107	157	81	154	53	127
P input (gP m^−2^ y^−1^)	87	99	92	115	55	78
P wasted (gP m^−2^ y^−1^)	25	46	38	46	22	37
Input fertilizer (gP m^−2^ y^−1^)	N/A	24	N/A	30	N/A	18
Mineral input CO_2_eq (kgCO_2_ m^−2^ y^−1^)	4.1	4.1	3.7	4.7	2.4	4.7
Squandered minerals CO_2_-eq (kgCO_2_ m^−2^ y^−1^)	1.06	1.7	0.59	1.7	0.38	2.0

Key performance indicators (KPIs) and resource flows are compared between IAA and DAP for each location under identical climatic conditions. B-IAA denotes the base configuration (internal waste streams only), whereas E-IAA denotes the manure-extended configuration with co-digestion. Values are annual averages normalized by hydroponic production system (HPS) area.

a Water loss via wet solids consists of unused wet solid material (digestate, fish sludge, plant waste). It is important to note that these streams were not included in the water use-efficiency (WUE) KPI because for the IAA networks they are negligible and distort the effect of zero-water discharge.

**Table 2:** Decision variables and constraints used in the dynamic optimal control problem

Symbol	Description	Unit	Base config	Extended config
$F_{dis,k}^{RAS}$	Ras water discharged to environment	[m^3^ d^−1^]	[0, 10]	[0, 100]
$F_{k}^{RAS\to HPS}$	Ras water sent to HPS	[m^3^ d^−1^]	[0, 10]	[0, 100]
$F_{dis,k}^{HPS}$	HPS water discharged to environment	[m^3^ d^−1^]	$\left[0,\frac{V_{HPS}}{5}\right]$	$\left[0,\frac{V_{HPS}}{5}\right]$
$M_{N_{fert},k}$	fertilizer (NO_3_ or TAN) supplemented to the HPS	[kg d^−1^]	[0, 10]	[0, 10]
$M_{P_{fert},k}$	fertilizer P supplemented to the HPS	[kg d^−1^]	[0, 5]	[0, 5]
$F_{manure}^{tot}$	diluted manure (5% Total solid level) used in AD1 and AD2	[m^3^ d^−1^]	*N*/*A*	[0, 0.4], [0, 0.35] [0, 0.8], [0, 0.5]

**System**	**Constraint**		**Unit**	**Description**

RAS	$0\leq C_{NO_{3}}^{RAS}\leq 200$		[mg L^−1^]	nitrate cap^16^
RAS	$C_{\mathrm{TAN}}^{RAS}\leq 4$		[mg L^−1^]	TAN cap^47^
HPS	$105\leq C_{N}^{HPS}\leq 250$		[mg L^−1^]	N target band^48^^,^^49^^,^^50^^,^^51^
HPS	$30\leq C_{P}^{HPS}\leq 56$		[mg L^−1^]	P target band^48^^,^^49^^,^^50^^,^^51^
AD1	1 ≤ *TS*_*AD*1,*in*_ ≤ 4		[%]	sludge TS feasibility^52^
AD2	1 ≤ *VS*_*AD*2,*in*_ ≤ 2		[g VS L^−1^ d^−1^]	volatile solid load feasibility^46^
AD1 & AD2	$F_{manure}^{tot}=F_{chick}+F_{cow}+F_{pig}$		[m^3^ d^−1^]	manure split equality
All	∑*F*_*in*,*k*_ = ∑*F*_*out*,*k*_		[m^3^ d^−1^]	closed volume balance

**Table 3:** Description of the key performance indicators (KPIs)

KPI	Mass balance description
WUE	$1-\frac{\sum_{k}\left(F_{dis,k}^{RAS}+F_{dis,k}^{HPS}\right)}{\sum_{k=1}^{N}F_{clean\;water,k}^{total,in}}$
NUE	$1-\frac{\sum_{k=1}\left(F_{dis,k}^{RAS}\cdot C_{N_{soluble},k}^{RAS}+F_{dis,k}^{HPS}\cdot C_{N_{soluble},k}^{HPS}+{M_{N}}_{digestate,k}\right)}{\sum_{k=1}\left(M_{N_{feed},k}+M_{N_{fert},k}+M_{N_{\;manure},k}\right)}$
PUE	$1-\frac{\sum_{k=1}\left(F_{dis,k}^{RAS}\cdot C_{P_{soluble}}^{RAS}+F_{dis,k}^{HPS}\cdot C_{P_{soluble}}^{HPS}+{M_{P}}_{digestate,k}\right)}{\sum_{k=1}\left(M_{P_{feed},k}+M_{P_{fert},k}+M_{P_{\;manure},k}\right)}$
NSS	$\frac{\sum_{k=1}\left(M_{N,k}^{RAS\to HPS}+M_{N,k}^{AD_{1}\to HPS}+M_{N,k}^{AD_{2}\to HPS}\right)\;}{\sum_{k=1}\left({M_{N,k}^{RAS\to HPS}+M_{N,k}^{AD_{1}\to HPS}+M_{N,k}^{AD_{2}\to HPS}+M}_{N_{fert},k}\right)}$
PSS	$\frac{\sum_{k=1}\left(M_{P,k}^{RAS\to HPS}+M_{P,k}^{AD_{1}\to HPS}+M_{P,k}^{AD_{2}\to HPS}\right)\;}{\sum_{k=1}\left({M_{P,k}^{RAS\to HPS}+M_{P,k}^{AD_{1}\to HPS}+M_{P,k}^{AD_{2}\to HPS}+M}_{P_{fert},k}\right)}$

KPIs allow to quantify water discharge requirements (WUE), squandering of nitrogen (NUE) and phosphorus (PUE) relative to total inputs. The numerator of the use-efficiency KPIs are losses and denominator are inputs. The amount of synthetic fertilizer relative to total inputs used in the hydroponic production system is measured using the nutrient self-sufficiency for nitrogen (NSS) and phosphorus (PSS). The numerator of the self-sufficiency is expressed as the nutrients originating from within the network over the total inputs.

DAP networks were optimized by selecting the hydroponic area and control trajectories under the constraint that only dissolved nutrients are available to the crop. Across all three locations, the optimizer converged to the same operating principle since the hydroponic greenhouse was sized such that nitrogen could be maintained close to the lower hydroponic N boundary. This limits dissolved-nitrogen losses and avoids water discharge from the aquaculture-hydroponic loop, but pushes P demand, which requires substantial supplementation (PSS = 8%).

Integrating anaerobic digestion within a RAS-HPS network enables recovery and reuse of nutrients contained in fish sludge and plant residues, alleviating phosphorus limitation and improving overall resource retention. Compared with DAP, wasted N decreased by 32% in Jakarta (157–107 gN m^−2^), 47% in Cairo (154–81 gN m^−2^), and 59% in Amsterdam (127–53 gN m^−2^), while wasted P decreased by 46% (46–25 gP m^−2^), 18% (46–38 gP m^−2^), and 40% (37–22 gP m^−2^), respectively. Consequently, the Jakarta base and extended configurations in Cairo and Amsterdam achieved full phosphorus self-sufficiency (PSS = 100%) without synthetic P additions, whereas DAP required 18–30 gP m^−2^ yr^−1^ of fertilizer (≈23%–26% of total P inputs). Total water inputs were also reduced in IAA by 23% in Jakarta (1237–955 L m^−2^), 7% in Cairo (1232–1140 L m^−2^), and 43% in Amsterdam (1284–730 L m^−2^). All optimized scenarios avoided liquid discharge. Remaining water loss occurred via wet solids (9.3–16.2 L m^−2^ in IAA, Table 1). In the manure-extended cases, mineral-input footprints decreased from 4.7 to 3.71 kgCO_2_-eq m^−2^ in Cairo and from 4.7 to 2.36 kgCO_2_-eq m^−2^ in Amsterdam, alongside large reductions in squandered minerals (−65% and −81%).

## Discussion

We treat an integrated recirculating aquaculture system (RAS) and hydroponic production system (HPS), coupled to anaerobic digestion, as a dynamic resource-allocation problem, in which time-varying, waste-derived nitrogen (N) and phosphorus (P) streams must be allocated to meet crop demand while maintaining fish- and crop water-quality constraints. By translating this coupled design-operation problem into a constrained optimal control problem, we position circular aqua-agriculture within the optimal control literature on constrained dynamic resource allocation. This connects our work to optimal-control studies in aquaculture, often focused on operational and economic decisions such as feeding regimes, stocking densities, harvesting, and system sizing^21^ and to agricultural optimal control, where applications range from minimizing actuator inputs while maximizing greenhouse climate conditions to efficient irrigation strategies.^22^ In the integrated setting, the aquaculture and hydroponic domains become coupled: operating policies that improve circularity (e.g., reduced discharge, higher nutrient reuse) can restrict feasible operating choices and change the best operating strategy, creating explicit trade-offs between resource conservation, operational robustness, and when economic objectives are included profitability.

The optimal-control problem that we have defined (Equations 1, 2, 3, 4, 5, 6, 7, and 8) is nonconvex, due to the nonlinear terms in the mass-balance equations where control flows are multiplied by time-varying concentration states. Therefore, the existence of a globally unique optimum cannot be analytically guaranteed. Instead, multiple local optima may exist due to nonlinearity and nonconvexity. The feasibility of the solution is ensured numerically by the fulfillment of the KKT conditions within the IPOPT solver. From a control-theoretic perspective, the feasibility of the “closed-loop” state is constrained by the controllability of the nutrient ratios. In the base configuration (digesters treating only fish sludge and plant residues), the lack of independent control over N and P mineralization can lead to two types of “trapped” states. The first type is a deficit which can only be solved via supplementation of synthetic fertilizer, while the second type involves oversaturation where discharge of nutrient-rich water becomes the only feasible action to prevent accumulation. The introduction of manure co-digestion in the extended configuration adds an independent control input, which allows adjustment of the N:P supply ratio and hence restores feasibility of discharge-free operation. Even when multiple local optima exist, the resulting trajectories are still physically consistent and align with the limits set by mass balances and operating constraints of the network.

Solving the optimal control problem separately for tropical, arid, and temperate climates shows that closed-loop operation in the base configuration is readily achievable under weak seasonality (Jakarta, Indonesia), becomes marginally P-limited under moderate seasonality (Cairo, Egypt), and requires discharge plus external synthetic fertilizers under strong seasonality due to N oversaturation and stronger P deficits (Amsterdam, the Netherlands). As seasonal variability increases, the network alternates more frequently between oversupply (cold, low-ET periods) and undersupply (warm, high-ET periods), making it harder to align waste-derived nutrient release with crop demand.

Differences between N and P biogeochemistry help explain why P-based indicators (e.g., PUE/PSS) are lower or more sensitive than N-based indicators as seasonality intensifies. N is largely (60%–90%) excreted by fish in dissolved form (TAN), which is readily available for plant uptake, while losses via nitrification-denitrification depend on oxygenation and process conditions.^6^ In contrast, P is predominantly retained in solids and must first be mineralized before becoming available to plants. Moreover, during organic-matter breakdown under near-neutral pH, released orthophosphate can be re-immobilized through precipitation or binding with metals (e.g., Fe, Mg), reducing the fraction that remains soluble and directly available for uptake.^13^^,^^23^^,^^24^ Together, variable mineralization and partial re-immobilization reduce the controllability of P relative to N, which can cause differences in N:P supply ratios and amplify seasonal supply-demand mismatches. In the present model, these processes are captured by a lumped mineralization coefficient and are not resolved individually. Disentangling the contributions of precipitation, microbial immobilization, and incomplete hydrolysis would require coupling biokinetic and geochemical speciation models, which remains an important direction for improving nutrient recovery predictions.

To address such imbalances, strategies such as desalination or buffer tanks have been proposed to concentrate nutrients or temporarily store and release them between low- and high-demand periods.^25^^,^^26^ Our optimization results and perturbation analysis suggest that such strategies can be omitted by adding an external mineralizable organic co-substrate to the digesters. When combined with an HPS sized beyond the internal waste-derived supply capacity, additional crop uptake helps consume nutrients during low-demand periods (reducing accumulation risk), while co-digestion supplies limiting nutrients during peak-demand periods.

In the manure-extended networks for Cairo and Amsterdam, co-digestion with livestock manure provided this additional flexibility and enabled closure of both N and P loops when combined with nutrient-rich RAS water. We considered three representative livestock manures with contrasting N:P ratios: chicken (3:1), pig (2.4:1), and cow (4.5:1).^27^ The optimizer selected predominantly pig manure in Cairo (85% of total input), whereas chicken manure was preferred in Amsterdam (71%). This difference originates from choosing an HPS area that matches the internal waste-derived nutrient supply during winter (or lowest evapotranspiration period). Subsequently, the optimal design in Amsterdam will require a larger HPS:RAS ratio compared to Cairo and is therefore more prone to nutrient deficits during summer (or highest evapotranspiration period). Under these conditions, the preferred co-substrate is the one that supplies sufficient mineralizable nutrients while keeping the N:P balance close to crop demand, which depends on both the N:P ratio and the amount of N and P supplied per unit of input under the evaluated bounds. This explains why chicken manure was favored over cattle manure in Amsterdam: despite cattle manure’s higher N:P ratio, it contributes less P relative to the required seasonal balance, so using it to cover peak-demand deficits would push the system toward N surplus. In Cairo, the required oversizing is smaller and P remains the dominant limiting nutrient, favoring a more P-targeted co-substrate such as pig manure. Overall, these results show that co-digestion is not only a way to address the mismatch between waste-derived nutrient supply and crop demand, but also a route to reduce external virgin inputs and nutrient-rich water losses under seasonal climates.

The perturbation analysis further shows how co-digestion can improve robustness against parameter uncertainty propagation between coupled systems. In the internal-waste-only baseline, under tropical conditions KPI sensitivity is mainly driven by evapotranspiration (demand) and FCR (supply). As seasonality increases, the feasible operating window narrows and parameters that cause negligible changes under tropical conditions can become influential. In the temperate case, for example, perturbations in fish excretion partitioning were sufficient to push N or P trajectories toward water-quality bounds, occasionally triggering discharge or external supplementation to remain feasible. Introducing manure adds an extra control layer that is tunable in flow, and to a certain extent in composition, and allows the optimizer to compensate for temporary shifts in under- and oversupply while keeping state constraints satisfied. In the manure-extended cases, this additional adjustable input was able to suppress KPI sensitivity (ΔWUE/ΔPSS ≈0 under ±20% perturbations) and therefore also reduced uncertainty propagation through the coupled mass balances. The errors originating in one system (e.g., mineralization coefficient, evapotranspiration) can subsequently be absorbed before cascading into downstream states and decisions. However, this improved robustness comes at the expense of additional capacity requirements (higher digester volume throughputs and larger HPS sizing relative to RAS).

To quantify how the additional flexibility of anaerobic digestion improves the network performance we compared it with optimized decoupled aquaponics networks (RAS-HPS). Across the three climate cases, integrating anaerobic digestion reduced nutrient losses substantially relative to optimized decoupled aquaponics (DAP). Wasted N decreased by around 46% on average (range: 32%–59%) and wasted P decreased by 35% on average (range: 18%–46%). In the best-performing integrated aqua-agriculture configurations, synthetic P inputs were eliminated and discharge was avoided, saving roughly 18–30 gP m^−2^ yr^−1^ relative to optimized DAP. This corresponded to an average 20% reduction in mineral-input CO_2_-eq (from virgin fertilizer manufacture) and a 60% reduction in squandered-minerals CO_2_-eq per unit of cultivated area.

Taken together, these reductions indicate that recovering nutrients from particulate waste streams, either internally generated (fish sludge and plant residues) or imported as a co-substrate, can substantially improve circular performance relative to decoupled aquaponics. The resource-efficiency gains identified in this study point to a larger global opportunity. A substantial share (often more than half for some species) of the N and P supplied in feed ultimately ends up in residual waste streams, across an estimated ∼58 million tons of fish feed used annually.^28^^,^^29^^,^^30^ Since aquaculture production is concentrated in many regions of the Global South (∼95% of global output),^30^^,^^31^ approaches that reduce reliance on virgin fertilizers by improving internal reuse may also strengthen resilience to input price volatility and help to improve food security. Beyond waste mitigation, fish-feed production itself poses environmental and economic challenges due to reliance on fishmeal and soybean meal. Within the network, valorizing the anaerobic digestate, the main remaining waste stream in our model, could further strengthen circularity. Options include conversion to insects (e.g., black soldier fly larvae), algae, or duckweed to recycle nutrients into feed,^32^ CO_2_ capture from biogas use for greenhouse enrichment, or bioplastic pathways from fish scales and plant residues.^33^^,^^34^^,^^35^ Such cascading use may be facilitated in eco-industrial food parks where firms co-locate and exchange by-products.^36^ Despite the pressing need, economic hurdles still hinder adoption of on-site waste-upcycling technologies. For example, it was found that large-scale anaerobic digestion of mixed farm residues in the Netherlands was unprofitable without subsidies, mainly due to transport costs and application limits that restricted fertilizer sales.^37^ In the integrated network proposed here, internal reuse can reduce disposal and fertilizer costs, but investment and operating costs remain non-trivial and profitability will likely depend on scale and logistics. However, looking forward, the economic tables might turn as resource scarcity and environmental policy instruments (e.g., carbon pricing or nutrient-emission regulation) could raise the cost of virgin fertilizers and feeds used in the aquaculture/livestock sector and incentivize circular resource recovery, potentially turning on-site anaerobic digestion from a cost burden into a competitive advantage.^38^^,^^39^^,^^40^

### Limitations of the study

It should be emphasized that this study is exploratory and primarily methodological. The main insights do not depend on precise parameter values, but on structural constraints, the admissible decision variables, and the optimization framework itself. Full validation at integrated-network scale remains challenging because large-scale coupled systems of the type modeled here are not yet widely available. Consequently, we rely on modular validation against literature-reported ranges for subsystem performance. Future pilot data would enable integrated validation and parameter identification, supporting site-specific engineering design and stronger uncertainty quantification.

Several modeling simplifications may nonetheless influence the quantitative optima. The anaerobic digesters are represented using zeroth-order mineralization kinetics rather than ADM1-level biochemical detail, thereby omitting intermediate metabolite dynamics, microbial community effects, and pathway-specific inhibition. As a result, the model cannot fully resolve the fate and form of phosphorus and nitrogen during digestion, and may misrepresent the timing and extent of nutrient release under co-digestion, as well as the scope for optimizing reactor operation with respect to inhibition risk (e.g., ammonia, volatile fatty acids [VFAs]), crop-specific VFA tolerance,^41^ and plant-available nutrient recovery. Similarly, the radiation- and evapotranspiration-driven crop model tends to overpredict lettuce yields by 10%–20% under high-radiation conditions, which could affect optimal system sizing. The sensitivity assessment is also scenario-based (±20% perturbations) rather than probabilistic, as parameter distributions for integrated operation are not yet available.

More broadly, the mass-balance KPIs do not capture greenhouse-gas emissions, logistics, storage losses, or residual organics in digestate. Life cycle and techno-economic assessment will be needed to translate resource-efficiency gains into net sustainability benefits. In addition, the loop-closing strategy presupposes controllable and capturable waste streams and is therefore most applicable to intensive or semi-closed production systems, whereas most global aquaculture still relies on ponds and open-water cages.^42^^,^^43^

## Resource availability

### Lead contact

Requests for further information and resources should be directed to and will be fulfilled by the lead contact, Xiaodong Cheng (xiaodong.cheng@wur.nl).

### Materials availability

This study did not generate new unique reagents.

### Data and code availability

• Data reported in this paper will be shared by the lead contact upon request.
• All original code is available in this paper’s supplemental information.
• Any additional information required to reanalyze the data reported in this paper is available from the lead contact upon request.

## Acknowledgments

This work is part of the BlueCycling project that has received funding from the European Union’s Horizon 2020 Research and Innovation Program under grant agreement no 862555 within the 2019 Joint Call of the ERA-NET Cofund on Food Systems and Climate.

## Author contributions

Conceptualization, M.d.K. and J.B.; methodology, M.d.K., J.B., L.G.v.W., and K.J.K.; investigation: M.d.K.; writing – original draft, M.d.K.; writing—review and editing, M.d.K., V.L., A.J., X.C., and K.J.K.; funding acquisition, K.J.K.; supervision, X.C. and K.J.K.

## Declaration of interests

The authors declare no conflict of interest (both financial and non-financial).

## STAR★Methods

### Key resources table

**Table:** 

REAGENT or RESOURCE	SOURCE	IDENTIFIER
**Software and algorithms**		

Jupyter lab	Project Jupyter	https://jupyter.org; v4.5.2
Python	Python Software Foundation	https://www.python.org; v3.11.13
Gekko	Beal et al.^44^	https://gekko.readthedocs.io; v1.3.0
Open-Meteo Weather API	Zippenfenig^45^	https://doi.org/10.5281/zenodo.7970649

### Experimental model and study participant details

This was a computational model-based study and did not involve live animals, plants, human participants, cell lines, microbial strains, or primary cell cultures. The biological species represented in silico were Nile tilapia (*Oreochromis niloticus*) and lettuce (*Lactuca sativa*); their simulated production settings, model assumptions, and parameter ranges are described under method details.

### Method details

#### Description of the networks

We model an industrial aqua-agriculture network that couples a 100 m^3^ recirculating aquaculture system (RAS) for Nile tilapia (*Oreochromis niloticus*) with a hydroponic production system (HPS) for lettuce (*Lactuca sativa*) and two anaerobic digesters (AD1 treating fish sludge; AD2 treating crop residues). The HPS cultivation area is a design variable, while the RAS volume is fixed. Irrigation water to the HPS is supplied by a controlled mixture of (i) RAS water transfer, (ii) digester effluents, and (iii) clean freshwater to satisfy the HPS volume balance. Water-quality constraints in the RAS and HPS are enforced by allocating flows between subsystems and, when required, discharging water from the RAS and/or HPS.

We consider two configurations that share the same network structure. In the base configuration, only internal waste streams (fish sludge and inedible crop residues) are digested. In the extended configuration, livestock manure can be co-digested to provide an additional mineralizable nutrient source and allow adjustment of the N:P supply ratio. Three type of manures (pig, chicken, cattle) with different N:P ratios (2.4:1; 3:1; 4.5:1)^27^ were used in the model and set as a decision variable. We allowed the model to select both single manures or as a mixture.

#### Modeling procedure

Previously published fish growth and greenhouse climate models were used to precompute the daily fish feed requirements and crop evapotranspiration rates. These values served as inputs for the mass balances describing water and nutrient dynamics in the aquaculture unit, anaerobic digesters, and hydroponic greenhouse. The ambient temperature, direct radiation, and humidity levels measured in Jakarta Cairo, and Amsterdam from 2022 were inputs to the greenhouse climate model.^45^

After determining these external inputs, we performed steady-state mass balance calculations and, where indicated, static feasibility/sizing optimizations to determine system dimensions and consistent initial conditions prior to dynamic optimization. The procedure for converting the dynamic model to steady-state mass balances is outlined in previous work.^17^ The steps for the base and extended configuration are summarized below.

##### Procedure base configuration

1a) Steady-state initialization

Using the starting fish biomass and corresponding feed input, a steady-state mass-balance calculation was performed to obtain consistent initial conditions in the aquaculture system and in the anaerobic digester treating fish sludge (AD1). Initial hydroponic nutrient concentrations were set to the midpoint of the lower and upper target bounds. The initial conditions in the plant-waste digester (AD2) were obtained from a steady-state calculation based on the average plant waste production implied by the mean evapotranspiration rate and a chosen hydroponic greenhouse area.

2a) Dynamic optimization and greenhouse area adjustment

Using these initial conditions, a dynamic optimal control problem (Section 2.3) was solved for a range of hydroponic greenhouse area sizes to identify configurations that did not require synthetic fertilizers while keeping nutrient concentrations below their upper bounds. This procedure was repeated iteratively (adjusting the greenhouse area) until the best feasible configuration was found.

##### Procedure extended configuration

For the extended network, a similar procedure was followed, with additional static optimization steps to determine feasible system dimensions and the required manure input.

1b) High-supply/low-demand (static optimization)

A steady-state optimization problem was solved with maximum fish feed input and minimum crop evapotranspiration to determine the hydroponic greenhouse area (and associated system dimensions) that keeps nutrient concentrations below their maximum thresholds under peak nutrient release from internal waste streams.

2b) Maximum manure input (static optimization)

Using the dimensions from step 1, we performed a steady-state optimization at the annual mean fish-feed and evapotranspiration inputs to determine a manure bound that avoids synthetic fertilizer use without over-sizing digester volumes. This bound was then verified in step 3 using the full-year dynamic optimal control problem under daily weather fluctuations.

3b) Initialization and dynamic optimization

With the hydroponic area and manure bound from steps 1b–2b, steady-state mass-balance calculations were used to compute consistent initial conditions at the start of the year. These initial conditions were then used in the dynamic optimal control problem to identify control trajectories that satisfy the constraints and optimize the performance index.

A schematic flow chart of this procedure is shown below in Figure 7.

#### Optimal control problem formulation

The industrial aqua-agriculture network model was developed in Python (v3.12.4) and optimized with the Interior Point Optimizer (IPOPT) algorithm, a nonlinear optimization solver available in the open source package GEKKO.^44^ We solved a dynamic optimal control problem over [*t*_0_, *t*_*f*_] to determine operational trajectories that satisfy the model dynamics and all path constraints while minimizing a scalar performance index *J*.

The network dynamics are described by a continuous-time nonlinear state-space model

(Equation 1) *dx*(*t*)/*dt* = *f*(*x*(*t*), *u*(*t*), *d*(*t*), *p*),  *x*(*t*_0_) = *x*_0_,  *t*_0_≤*t* ≤ *t*_*f*_

with the augmented state vector $x\left(t\right)\in R^{n_{x}+1}$, control vector $u\left(t\right)\in R^{n_{u}}$, disturbance vector $d\left(t\right)\in R^{n_{d}}$, and parameter vector $p\in R^{n_{p}}$.

Here, *n*_*x*_ denotes the number of physical/chemical state variables, including nitrate, TAN, solid N, soluble P and solid P concentrations in the RAS and both digesters, and nitrate, TAN and soluble P in the HPS, while the additional state dimension $x_{n_{x}+1}=J\left(t\right)$ is the augmented objective state used to transform the Lagrange functional into Mayer form (Equation 7).

The controls *u*(*t*) are the daily flow allocations/discharges and external additions (fertilizer and, when enabled, manure), while *d*(*t*) contains the daily fish feed and evapotranspiration rate profiles.

The continuous-time control and disturbance variables are parameterized at daily resolution and held piecewise constant between updates, i.e.,

$$u\left(t\right)=u_{k},d\left(t\right)=d_{k},t\in [t_{k},t_{k+1}),\;k=0,1,\ldots ,N-1$$

The admissible state and control trajectories are constrained to lie in the sets $X\subset R^{n_{x}+1}$ and $U\subset R^{n_{u}}$ i.e.,

(Equation 2) $$x\left(t\right)\in X,\;u\left(t\right)\in U,\;\forall \;t\in \left[t_{0},t_{f}\right]$$

In the Lagrange form, the performance index is defined as

(Equation 3) $$J\left(u\right)=\int_{t_{0}}^{t_{f}}L\left(x\left(t\right),u\left(t\right),t\right)\;dt,\;J,L\in R^{1}$$

Where L is the running cost. The optimal control problem is then,

(Equation 4) $$\min_{u\left(\cdot \right)\in U}J\left(u\right)$$

subject to the dynamic constraints (Equation 1), the admissible state and control sets (Equation 2) and the path constraints

(Equation 5) $$g\left(x\left(t\right),u\left(t\right),t\right)\leq 0,\;t_{0}\leq t\leq t_{f}$$

(Equation 6) $$h\left(x\left(t\right),u\left(t\right),t\right)=0,\;t_{0}\leq t\leq t_{f}$$

Where *g*(·) and *h*(·) are vector-valued nonlinear inequality and equality constraint functions of appropriate dimensions.

For numerical implementation in GEKKO, the Lagrange formulation (Equation 3) was rewritten into an equivalent Mayer form by augmenting the state vector with an extra objective state *J*(*t*), with dynamics

(Equation 7) $$\dot{J}\left(t\right)=L\left(x\left(t\right),u\left(t\right),d\left(t\right),t\right),\;J\left(t_{0}\right)=0$$

So that minimizing *J*(*u*) is equivalent to minimizing the terminal value *J*(*t*_*f*_).

In this study, the cost function includes (i) penalties on external fertilizer use and water discharge, (ii) tracking terms that penalize deviations of nutrient states from their targets over the year, and (iii) a regularization term that keeps the volatile-solids loading rate close to its optimal operating point.

(Equation 8) $$\dot{J}\left(t\right)=\left(w_{1}\Phi_{flow}\left(t\right)+{w_{2}\Phi }_{track}\left(t\right)+w_{3}\Phi_{VS}\left(t\right)\right)$$

$$\Phi_{flow}=M_{N,fert}\left(t\right)+M_{P,fert}\left(t\right)+F_{dis}^{RAS}\left(t\right)+F_{dis}^{HPS}\left(t\right)$$

$${\Phi_{track}=\frac{\left(C_{i}^{HPS}\left(t\right)-C_{i,set}^{HPS}\right)}{C_{i,set}^{HPS}}}^{2}+\frac{{\left(C_{NO_{3}}^{RAS}\left(t\right)-C_{i,set}^{RAS}\right)}^{2}}{C_{i,set}^{HPS}}$$

$$\Phi_{VS}={\left(\frac{VS_{LR}^{AD_{2}}\left(t\right)-VS_{opt}}{VS_{opt}}\right)}^{2}$$

Here *M*_*TAN*_, *MNO*_3_, and *M*_*P*_ [kg d^−1^] denote the daily masses of ammonium-, nitrate-, and phosphorus-containing fertilizers added to the irrigation water, $F_{dis}^{RAS}$ and $F_{dis}^{HPS}$ [m^3^ d^−1^] denote the daily discharge volumes from the aquaculture and hydroponic units. Moreover, $C_{i}^{HPS}\left(t\right)$ [mg L^−1^] is the hydroponic concentration of nutrient *i*∈{*N*,*P*}, and $C_{i,set}^{HPS}$ is its setpoint (set equal to $C_{i}^{HPS}\left(t_{0}\right)$). The weighing factors $w_{1-3}>0$, were tuned to balance the relative magnitudes of the penalty terms, if necessary.

The objective function retained the same general structure across all scenarios. The tracking and VS-loading regularization terms were activated only in the extended (manure) configuration ($w_{2},w_{3}>0$) because manure addition introduces additional degrees of freedom for nutrient tuning and therefore benefits from explicit regularization to keep the AD2 volatile-solids loading rate close to the vegetable-waste optimum.^46^ In the base configuration, these terms were set to zero ($w_{2}=w_{3}=0$).

Feasibility of the optimal control solution was enforced through the bounds on the decision variables and the path and balance constraints summarized in Table 2, which define admissible water-quality ranges for the RAS and HPS and operational feasibility limits for the digesters.

For each scenario, the resulting optimal trajectories were evaluated using annual key performance indicators (KPIs) that quantify water discharge, nutrient-use efficiency, and nutrient self-sufficiency. A generic description of the states describing the water quality in each system is given in Equation 9 and a description of each KPI is provided in Table 3. The full set of state equations, parameters are provided in the supplementary information (Equations S1–S40).

Each concentration state follows a generic mass balance of the form

(Equation 9) $${{\dot{C}}_{s}}^{j}\left(t\right)=\frac{\sum_{k}Q_{k\to j}\left(t\right)\;C_{s}^{k}\left(t\right)-C_{s}^{j}\left(t\right)\sum_{l}Q_{j\to l}\left(t\right)+P_{s}^{j}\left(t\right)-U_{s}^{j}\left(t\right)}{V_{j}}$$

here, the first sum is taken over all compartments *k* that feed into compartment *j*, while the second sum is taken over all compartments *l* receiving outflow from *j*. *V*_*j*_ is the compartment volume and $P_{s}^{j}$ and $U_{s}^{j}$ are the production and uptake terms (e.g., nitrification, plant uptake) of species *s* in compartment *j*.

#### Model description and validation

Validating our network model remains challenging since no large-scale integrated food production network of this kind currently exists. Nevertheless, in what follows we validated each system component contributing to the entire network. A short description of each system and validation results are presented in the following sections. For more technical details regarding all sub-models and a list of parameters we refer to Tables S1–S3. This modular validation approach focuses on verifying the integrated model using the production and operational outputs (fish yield, aquaculture water exchange rates, chemical oxygen demand removal in the digesters, and lettuce biomass production). Specifically, we examine whether the integrated model produces outcomes that are consistent with values reported in aquaculture and hydroponic literature.

In addition to this, we investigate uncertainty propagation in the coupled models. The integrated model couples the RAS, HPS, and AD sub-models through conserved water, nitrogen, and phosphorus mass balances, such that outputs of one system (e.g., nutrient loads and flows) become inputs to the next. We verified dynamic coupling by checking annual mass-balance closure and by confirming that all internal states and operating variables remain within the validity ranges reported for the source models (e.g., RAS/HPS water-quality bounds, AD TS/VS loading feasibility ranges and constant hydraulics). To assess how uncertainty propagates through these couplings, we performed a scenario-based sensitivity analysis in which key parameters are perturbed by ±20% and the full dynamic optimization problem is re-solved for each perturbation. Any resulting changes in state trajectories that exceed constraints and are corrected using discharge/fertilizer decisions are directly measurable by the KPIs that quantify the system-level effect of parameter uncertainty transmitted through the coupled mass balances.

#### The recirculating aquaculture system and fish growth model

The recirculating aquaculture system (RAS) was assumed to be a well-managed in terms of temperature (29°C), optimal DO levels, and high quality feed. The RAS consisted of ten tanks with a combined volume of 100 m^3^, where Tilapia were reared in cohorts over a 252-day growth cycle at a maximum stocking density of 80 kg m^−3^ or a harvest target density of 600 g fish^−1^. This is in line with conventional tilapia harvest weights of 600–900 g in 5–8 months when starting from fingerlings (10–30 g).^53^ Total harvested mass in our model resulted in an annual yield of 11318 kg of tilapia with an average stocking density of 22 kg m^−3^. Feed requirements were determined using an empirical temperature driven growth model and FCR model, validated in previous research.^54^ Feed consumption and fish metabolism were modeled using zeroth-order reactions and assumed to occur within a one-day time step. The optimized stand-alone aquaculture unit kept nitrate (≤200 mg L^−1^) and total ammonia nitrogen (≤4 mg L^−1^) below toxic levels by exchanging 1.5% of the water volume, which is near the range of 0.8–1.1% reported in the literature.^55^^,^^56^ The water flows and nutrient concentrations are found in Data S1 and described by Equations S1–S9.

#### The anaerobic digestion model

We represented the anaerobic digesters as simplified continuously stirred tank reactors (CSTRs) with zeroth-order mineralization kinetics, rather than using the full Anaerobic Digestion Model No. 1 (ADM1), because our focus was nutrient mineralization for reuse in the hydroponic loop rather than detailed biogas and intermediate volatile fatty acid dynamics. Two digesters were included: AD1 treated fish sludge and was operated at a total solids (TS) content of 1.5–2.0%, while AD2 treated lettuce crop residues and was designed to operate within a feasible wet-digestion volatile solids (VS) loading range of 1–2 g L^−1^ d^−1^, with a nominal target of 1.5 g L^−1^ d^-1.^^46^

In the manure-extended network configuration, both digesters could be co-fed with livestock manure to provide additional mineralizable nutrients and operational flexibility. Despite livestock manure typically having a low C:N ratio, previous studies have shown that this does not compromise reactor stability once the inoculum is properly acclimated.^13^^,^^24^ In our model set-up, manure was diluted to 5% TS level to prevent ammonia inhibition^18^ and co-feeding was constrained such that the combined digester feed remained within TS ≤ 3.0–3.5%, consistent with reported operational ranges for fish-sludge digestion.^52^ Co-digestion also helped maintain feasible VS loading during periods when lettuce residue inputs were low or highly variable.

Because quantitative data on N and P mineralization from co-digested manures under fish-sludge/vegetable-residue conditions are limited, we assumed that manure mineralization behavior was comparable to fish-sludge digestion. This simplifying assumption should be evaluated experimentally, particularly for cattle manure, which typically contains more fiber/lignocellulosic material and may hydrolyze more slowly than poultry or pig manure, potentially affecting both nutrient release and biogas yields.^57^

Finally, mineralization coefficients and operational bounds (e.g., VS loading and HRT) were treated as constant over the simulated year, implying stable conversion performance. In practice, microbial community composition and activity can shift with substrate mixtures, temperature, inhibitory compounds (e.g., ammonia), and trace-element availability, which may alter hydrolysis and methanogenesis rates over longer timescales. Long-term stability under fish-sludge and vegetable substrates has been reported after start-up in controlled experiments.^58^^,^^59^ Nevertheless, explicitly coupling microbial dynamics or adaptive kinetics to digestion performance, especially under variable co-digestion mixtures, remains an important direction for future work.

The reactors were operated at hydraulic retention times of 30 and 25 days, respectively, following earlier experimental studies on fish-sludge and vegetable-waste digestion.^13^^,^^52^ These studies did not report instability associated with secondary microbial by-products such as ammonia and volatile fatty acids, suggesting that these HRTs represent plausible operating values for the present system-level analysis. Although the digesters were represented using zeroth-order mineralization kinetics, we additionally considered first-order hydrolysis constants reported in the literature as an external plausibility check on whether the selected HRTs are consistent with reported substrate-specific conversion timescales. Based on a first-order hydrolysis constant of 0.5 days^−1^, the selected HRT range of 25–30 days would correspond to approximately 92–94% hydrolysis of fish-sludge solids in an equivalent CSTR formulation.^60^ For lettuce residues, however, Yan et al.^61^ reported a lower hydrolysis rate constant (k = 0.11 days^−1^) in batch experiments. If translated to an equivalent CSTR-based HRT requirement, this would imply that approximately 80 days may be required to achieve more than 90% hydrolysis. This is considerably longer than the 25-day HRT used in our simulations for the plant-waste digester, which was based on semi-continuous pilot-scale digestion of vegetable waste reporting 90% VS removal within 25 days.^46^ Together, these findings illustrate that hydrolysis performance is strongly dependent on substrate characteristics, inoculum, and reactor configuration, and that the required HRT, and thus reactor sizing, may differ from the values used here. However, such differences are expected to affect reactor volume and associated costs more strongly than the qualitative closed-loop nutrient-cycling conclusions of the present study.

Reactor volumes remained constant by adding fresh water when the total inflow of sludge water and manure or incoming crop fresh weight with manure was less than the outgoing flow. The required volume of both reactors were determined by their maximum allowable volumetric peak loads since the organic loading rates stayed below the maximum organic loading rates of 20–27 kg COD m^−3^ day^−1^ reported for reactors operating at the mesophilic optimum temperature of 40°C.^62^ The total ammonium nitrogen (TAN) levels stayed below the mesophilic limit of 3 g L^-1.^^63^^,^^64^ Furthermore, the mass and energy balances describing biogas production and the energy required to regulate reactor temperatures indicated an energy yield of approximately 1.4 kWh kgCOD^−1^, which aligns with values reported for large-scale commercial anaerobic digestion systems. Mass balances describing the water flows and nutrient concentrations and energy balances are found in Data S1 and described by Equations S10–S33.

#### The hydroponic production system and climate model

The hydroponic production system was equipped with 18 lettuce plants m^−2^ growing in a staggered production cycle of 50 days, with growth estimated from nitrogen assimilation dependent on the evapotranspiration rate and nitrogen concentration. The daily evapotranspiration rate was calculated using a revised Penman-Monteith equation, which takes into account the leaf area index.^65^ A leaf area index described for a standard loose-leaf lettuce variety using the same planting density with an initial and maximum index of 6.5 cm^2^ m^−2^ and 3 m^2^ m^−2^ was used to describe the plant cover.^66^^,^^67^ Microclimate interactions and greenhouse conditions were simulated using a validated Venlo-type greenhouse climate model using the ambient temperature, humidity, and radiation.^25^ Important to note, is that artificial lighting (200 W lamps) was used in the greenhouse model when computing the evapotranspiration rates for the temperate climate case study, which is standard practice in the Netherlands. Combining these choices led to average plant growth rates, expressed on a fresh-weight basis, of 8.8 g d^−1^ in Jakarta, 9.6 g d^−1^ in Cairo, and 6.2 g d^−1^ in Amsterdam. The computed growth rates in the tropical and arid climate were slightly above the typical 5–8 g d^−1^ range observed in hydroponic and aquaponic experiments.^68^^,^^69^^,^^70^ While these studies did not grow lettuce hydroponically under the same conditions as our simulation, potential factors resulting in the slightly higher yields under higher radiation conditions can be explained by the model. Firstly, the Penman-Monteith method used in the greenhouse climate sub-model tends to overestimate yield by combining evaporation and transpiration effects.^71^ Secondly, the mass balance describing plant growth was not equipped with a logistic growth term that slows down the growth rates at higher nitrogen concentrations. Adjusting our model by refining the crop coefficient to separate evaporation from transpiration, and including a logistic growth term, will likely result in production values closer to field observations. Overestimating lettuce production makes our estimates of self-sufficiency conservative, as it implies that actually less waste is produced per square meter of planting area. Important to note is that the crop growth rates found for the decoupled aquaponics networks were within the typical ranges (5.5–5.9 g d^−1^) because these networks tended to operate near the low nitrogen boundary. Comparing these rates with the integrated aqua-agriculture networks suggests that overestimating lettuce yields can mainly be attributed to the higher nitrogen content. Mass balances describing the water flows and nutrient concentrations in the hydroponic production system are found in Data S1 and described by Equations S37–S40.

### Quantification and statistical analysis

No statistical analyses were performed because this study is a computational, model-based optimization study and did not involve experimental group comparisons, biological replicates, or hypothesis testing. Quantitative results were obtained from deterministic model simulations and static/dynamic optimization runs. Model performance was evaluated using the key performance indicators defined in the method details section, including water use efficiency, nitrogen and phosphorus use efficiency, and nitrogen and PSS. The model was implemented in Python v3.12.4 and optimized using GEKKO with IPOPT, as described in the method details section.

## Supplemental information

Document S1. Figures S1–S7, Tables S1–S3, Methods S1 and S2, and supplemental references

## References

[1] Richardson K., Steffen W., Lucht W., Bendtsen J., Cornell S.E., Donges J.F., Drüke M., Fetzer I., Bala G., Von Bloh W. (2023). Earth beyond six of nine planetary boundaries. Sci. Adv..

[2] Independent Group of Scientists appointed by the Secretary-General (2023). Global Sustainable Development Report 2023: times of crisis, time of change: science for accelerating transformations to sustainable development (United Nations).

[3] Yep B., Zheng Y. (2019). Aquaponic trends and challenges–A review. J. Clean. Prod..

[4] Ritchie H. (2019). https://ourworldindata.org/rise-of-aquaculture.

[5] Garlock T., Asche F., Anderson J., Bjørndal T., Kumar G., Lorenzen K., Ropicki A., Smith M.D., Tveterås R. (2020). A Global Blue Revolution: Aquaculture Growth Across Regions, Species, and Countries. Rev. Fish. Sci. Aquac..

[6] van Rijn J. (2013). Waste treatment in recirculating aquaculture systems. Aquac. Eng..

[7] Montanhini Neto R., Ostrensky A. (2015). Nutrient load estimation in the waste of Nile tilapia *Oreochromis niloticus* (L.) reared in cages in tropical climate conditions. Aquac. Res..

[8] Paredes D., Kuschk P., Mbwette T.S.A., Stange F., Müller R.A., Köser H. (2007). New Aspects of Microbial Nitrogen Transformations in the Context of Wastewater Treatment – A Review. Eng. Life Sci..

[9] Rakocy J.E., Tidwell J.H. (2012). Aquaculture Production Systems.

[10] Neori A., Krom M.D., Rijn J.v. (2007). Biogeochemical processes in intensive zero-effluent marine fish culture with recirculating aerobic and anaerobic biofilters. J. Exp. Mar. Biol. Ecol..

[11] Goddek S., Delaide B.P.L., Joyce A., Wuertz S., Jijakli M.H., Gross A., Eding E.H., Bläser I., Reuter M., Keizer L.C.P. (2018). Nutrient mineralization and organic matter reduction performance of RAS-based sludge in sequential UASB-EGSB reactors. Aquac. Eng..

[12] Khalid A., Arshad M., Anjum M., Mahmood T., Dawson L. (2011). The anaerobic digestion of solid organic waste. Waste Manag..

[13] Zhu Z., Yogev U., Keesman K.J., Gross A. (2021). Onsite anaerobic treatment of aquaponics lettuce waste: digestion efficiency and nutrient recovery. Aquac. Int..

[14] Zhu Z., Keesman K.J., Yogev U., Gross A. (2022). Onsite anaerobic treatment of tomato plant waste as a renewable source of energy and biofertilizer under desert conditions. Bioresour. Technol. Rep..

[15] Dijkgraaf K.H., Goddek S., Keesman K.J. (2019). Modeling innovative aquaponics farming in Kenya. Aquac. Int..

[16] Goddek S., Körner O. (2019). A fully integrated simulation model of multi-loop aquaponics: A case study for system sizing in different environments. Agric. Syst..

[17] de Korte M., Bergman J., van Willigenburg L.G., Keesman K.J. (2024). Towards a zero-waste aquaponics-centered eco-industrial food park. J. Clean. Prod..

[18] Bi S., Westerholm M., Qiao W., Xiong L., Mahdy A., Yin D., Song Y., Dong R. (2020). Metabolic performance of anaerobic digestion of chicken manure under wet, high solid, and dry conditions. Bioresour. Technol..

[19] Firman T., Surbakti I.M., Idroes I.C., Simarmata H.A. (2011). Potential climate-change related vulnerabilities in Jakarta: Challenges and current status. Habitat Int..

[20] Fouad S.S., Heggy E., Ramah M., Abotalib A.Z., Palmer E.M., Jomaa S., Weilacher U. (2023). Egypt’s waterways conservation campaigns under growing intrinsic demand and Nile upstream damming. J. Hydrol.: Reg. Stud..

[21] Domínguez-May R., Hernández J.M., Velázquez-Abunader I. (2024). A review of dynamic optimization in aquaculture production economics. Rev. Aquac..

[22] Ding Y., Wang L., Li Y., Li D. (2018). Model predictive control and its application in agriculture: A review. Comput. Electron. Agric..

[23] Liu J., Cheng X., Qi X., Li N., Tian J., Qiu B., Xu K., Qu D. (2018). Recovery of phosphate from aqueous solutions via vivianite crystallization: Thermodynamics and influence of pH. Chem. Eng. J..

[24] Yogev U., Vogler M., Nir O., Londong J., Gross A. (2020). Phosphorous recovery from a novel recirculating aquaculture system followed by its sustainable reuse as a fertilizer. Sci. Total Environ..

[25] Jansen L., Keesman K.J. (2022). Exploration of efficient water, energy and nutrient use in aquaponics systems in northern latitudes. Clean. Circ. Bioeconomy.

[26] Goddek S., Keesman K.J. (2018). The necessity of desalination technology for designing and sizing multi-loop aquaponics systems. Desalination.

[27] CBAV (2025). Composition of different types of livestock manure. Handb. Bodem En Bemest.

[28] Fry J.P., Mailloux N.A., Love D.C., Milli M.C., Cao L. (2018). Feed conversion efficiency in aquaculture: do we measure it correctly?. Environ. Res. Lett..

[29] Tacon A.G.J., Metian M. (2015). Feed Matters: Satisfying the Feed Demand of Aquaculture. Rev. Fish. Sci. Aquac..

[30] FAO (2025). https://www.fao.org/fishery/en/statistics/software/fishstatj.

[31] FAO (2024).

[32] Pinho S., Leal M.M., Shaw C., Baganz D., Baganz G., Staaks G., Kloas W., Körner O., Monsees H. (2024). Insect-based fish feed in decoupled aquaponic systems: Effect on lettuce production and resource use. PLoS One.

[33] Zhu Z., Pinho S.M., Körner O., Turchini G.M., Lopes I.G., Brown P.B., Ye Z., Zhao J., Luo G., Monsees H. (2026). On how to make aquaponics more circular. Bioresour. Technol..

[34] Chang J.M., Joye I.J. (2024). Improving agricultural sustainability – A review of strategies to valorize tomato plant residues (TPR). Waste Manag..

[35] Thammahiwes S., Riyajan S.-A., Kaewtatip K. (2017). Preparation and properties of wheat gluten based bioplastics with fish scale. J. Cereal. Sci..

[36] Heeres R.R., Vermeulen W.J.V., De Walle F.B. (2004). Eco-industrial park initiatives in the USA and the Netherlands: first lessons. J. Clean. Prod..

[37] Gebrezgabher S.A., Meuwissen M.P.M., Prins B.A.M., Lansink A.G.J.M.O. (2010). Economic analysis of anaerobic digestion—A case of Green power biogas plant in The Netherlands. NJAS Wagening. J. Life Sci..

[38] Klenert D., Mattauch L., Combet E., Edenhofer O., Hepburn C., Rafaty R., Stern N. (2018). Making carbon pricing work for citizens. Nat. Clim. Chang..

[39] Liu X., Elgowainy A., Wang M. (2020). Life cycle energy use and greenhouse gas emissions of ammonia production from renewable resources and industrial by-products. Green Chem..

[40] Cordell D., White S. (2015). Tracking phosphorus security: indicators of phosphorus vulnerability in the global food system. Food Secur..

[41] de Korte M., Pinho S.M., Keesman K.J., Körner O. (2025). Cultivar-specific responses of hydroponically grown lettuce to volatile fatty acids: implications for using anaerobic digester effluents as organic fertilizers. J. Agric. Food Res..

[42] Small B.C., Hardy R.W., Tucker C.S. (2016). Enhancing fish performance in aquaculture. Anim. Front..

[43] Xiao R., Wei Y., An D., Li D., Ta X., Wu Y., Ren Q. (2019). A review on the research status and development trend of equipment in water treatment processes of recirculating aquaculture systems. Rev. Aquac..

[44] Beal L.D.R., Hill D.C., Martin R.A., Hedengren J.D. (2018). GEKKO Optimization Suite. Processes.

[45] Zippenfenig P. (2023).

[46] Babaee A., Shayegan J. (2011). World Renewable Energy Congress – Sweden.

[47] El-Shafai S.A., El-Gohary F.A., Nasr F.A., Van Der Steen N.P., Gijzen H.J. (2004). Chronic ammonia toxicity to duckweed-fed tilapia (*Oreochromis niloticus*). Aquaculture.

[48] Mattson N.S., Peters C. (2014). A recipe for hydroponic success. Grow.

[49] Sapkota S., Sapkota S., Liu Z. (2019). Effects of Nutrient Composition and Lettuce Cultivar on Crop Production in Hydroponic Culture. Horticulturae.

[50] Resh H.M. (2022).

[51] Hoagland D.R., Arnon D.I. (1950).

[52] Mirzoyan N., Tal Y., Gross A. (2010). Anaerobic digestion of sludge from intensive recirculating aquaculture systems: Review. Aquaculture.

[53] FAO (2009). *Oreochromis niloticus*. Cult. Aquat. Species Fact Sheets. https://www.fao.org/fishery/docs/CDrom/aquaculture/I1129m/file/en/en_niletilapia.htm.

[54] Ebeling J.M., Timmons M.B., Tidwell J.H. (2012). Aquaculture Production Systems.

[55] Yogev U., Sowers K.R., Mozes N., Gross A. (2017). Nitrogen and carbon balance in a novel near-zero water exchange saline recirculating aquaculture system. Aquaculture.

[56] Martins C.I.M., Ochola D., Ende S.S.W., Eding E.H., Verreth J.A.J. (2009). Is growth retardation present in Nile tilapia Oreochromis niloticus cultured in low water exchange recirculating aquaculture systems?. Aquaculture.

[57] Nasir I.M., Mohd Ghazi T.I., Omar R. (2012). Anaerobic digestion technology in livestock manure treatment for biogas production: A review. Eng. Life Sci..

[58] Zhu Z., Yogev U., Goddek S., Yang F., Keesman K.J., Gross A. (2022). Carbon dynamics and energy recovery in a novel near-zero waste aquaponics system with onsite anaerobic treatment. Sci. Total Environ..

[59] Zhu Z., Yogev U., Keesman K.J., Gross A. (2024). Promoting circular economy: Comparison of novel coupled aquaponics with anaerobic digestion and conventional aquaponic systems on nutrient dynamics and sustainability. Resour. Conserv. Recycl..

[60] Choudhury A., Lepine C., Good C. (2023). Methane and Hydrogen Sulfide Production from the Anaerobic Digestion of Fish Sludge from Recirculating Aquaculture Systems: Effect of Varying Initial Solid Concentrations. Fermentation.

[61] Yan H., Zhao C., Zhang J., Zhang R., Xue C., Liu G., Chen C. (2017). Study on biomethane production and biodegradability of different leafy vegetables in anaerobic digestion. AMB Express.

[62] Van Lier J.B., Mahmoud N.A., Zeeman G., Henze M., van Loosdrecht M.C.M., Ekama G.A., Brdjanovic D. (2008). Biological Wastewater Treatment: Principles, Modelling and Design.

[63] Park S., Cui F., Mo K., Kim M. (2016). Mathematical models and bacterial communities for ammonia toxicity in mesophilic anaerobes not acclimated to high concentrations of ammonia. Water Sci. Technol..

[64] Gebauer R., Eikebrokk B. (2006). Mesophilic anaerobic treatment of sludge from salmon smolt hatching. Bioresour. Technol..

[65] Stanghellini C. (1987). Transpiration of Greenhouse Crops: An Aid to Climate Management.

[66] Tei F. (1996). Growth of Lettuce, Onion, and Red Beet. 1. Growth Analysis, Light Interception, and Radiation Use Efficiency. Ann. Bot..

[67] Graamans L., Van Den Dobbelsteen A., Meinen E., Stanghellini C. (2017). Plant factories; crop transpiration and energy balance. Agric. Syst..

[68] Touliatos D., Dodd I.C., McAinsh M. (2016). Vertical farming increases lettuce yield per unit area compared to conventional horizontal hydroponics. Food Energy Secur..

[69] Sublett W.L., Barickman T.C., Sams C.E. (2018). The Effect of Environment and Nutrients on Hydroponic Lettuce Yield, Quality, and Phytonutrients. Horticulturae.

[70] Delaide B., Goddek S., Gott J., Soyeurt H., Jijakli M. (2016). Lettuce (Lactuca sativa L. var. Sucrine) Growth Performance in Complemented Aquaponic Solution Outperforms Hydroponics. Water.

[71] Doorenbos J., Kassam A.H. (1979).

