# Supplementary material for: Climate dependent feasibility of closing water and nutrient cycles in industrial aqua-agriculture
Source: iScience. 2026 Jun 8;29(6):116266. doi: 10.1016/j.isci.2026.116266 (PMC13264035; doi:10.1016/j.isci.2026.116266)
Supplement: Document S1. Figures S1–S7, Tables S1–S3, Methods S1 and S2, and supplemental references [file mmc1.pdf]

iScience, Volume *29*

Supplemental information

Climate dependent feasibility of closing water and nutrient cycles in industrial aqua-agriculture

Milan de Korte, Joris Bergman, L. Gerard van Willigenburg, Victor Lobanov, Alyssa Joyce, Xiaodong Cheng, and Karel J. Keesman

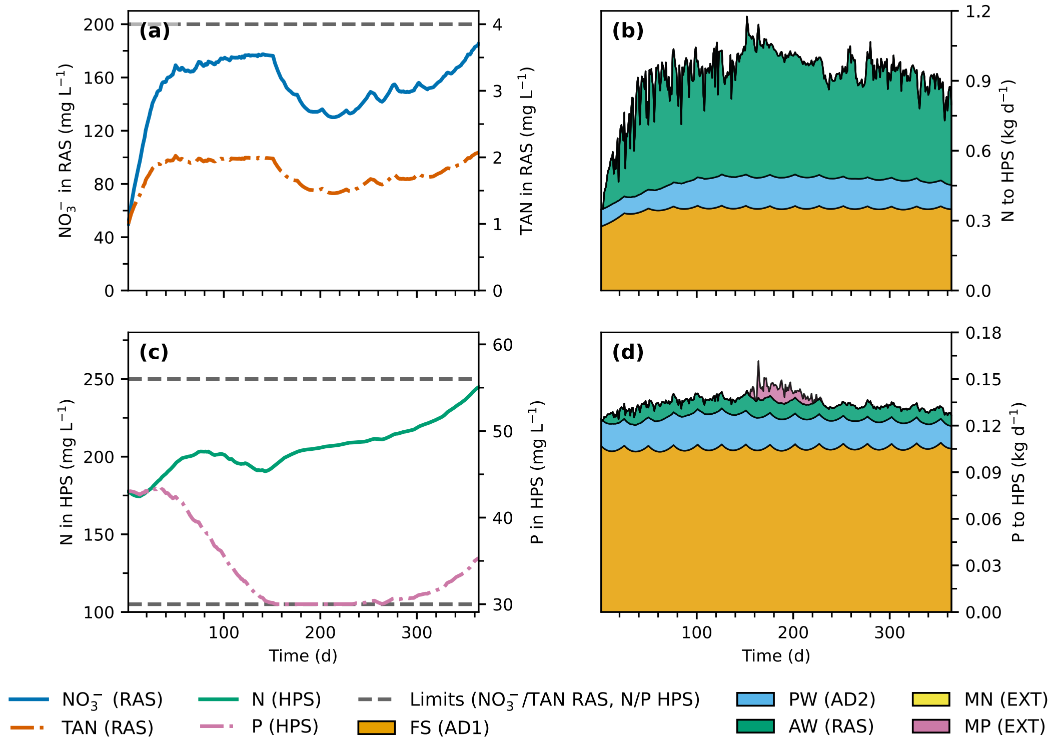

Figure S1 nutrient profiles of the integrated aqua-agriculture network in Cairo, related to Figure 2. RAS and HPS nutrient profile in base configuration of the industrial aqua-agriculture network in Cairo with an optimal HPS cultivation area of 1350 m^2. P becomes limiting and requires external supplementation as can be observed from subplot (d).

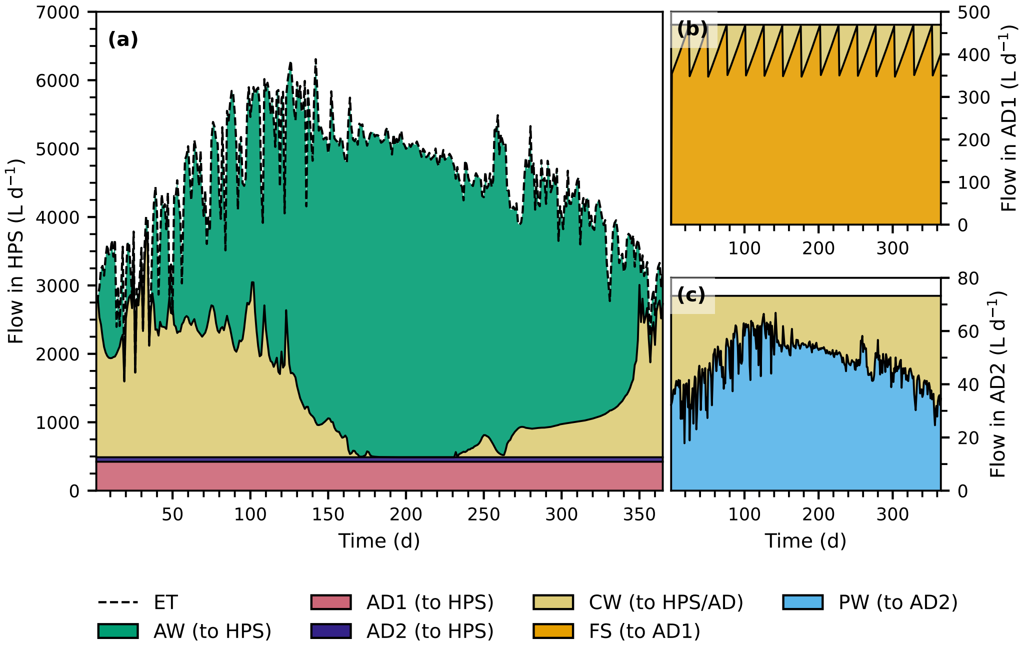

Figure S2 water flows of the integrated aqua-agriculture network in Cairo, related to Figure 3. Subplot (a) shows the water flows towards the HPS from two anaerobic digesters, one digesting fish sludge (AD1) and the other plant waste (AD2), and nutrient-rich water from the recirculating aquaculture system (AW). These flows align with the greenhouse evapotranspiration (ET) in, denoted by the black dashed-dotted line. Subplots (b) and (c) show the incoming anaerobic digester flows consisting of sludge water (SW), plant waste (PW), and additional clean water (CW1, CW2).

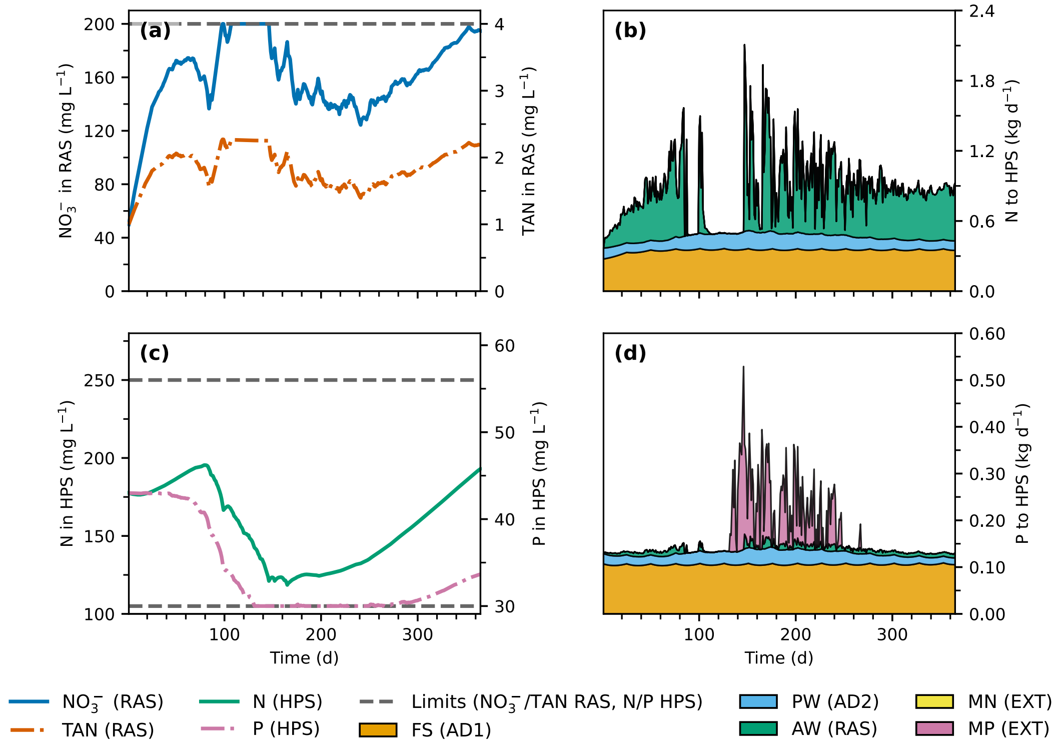

Figure S3 nutrient profiles of the integrated aqua-agriculture network in Amsterdam, related to Figure 2. RAS and HPS nutrient profile in base configuration of the industrial aqua-agriculture network in Amsterdam with an optimal HPS cultivation area of 2700 m^2. P becomes limiting and requires supplementation as can be observed from subplot (d).

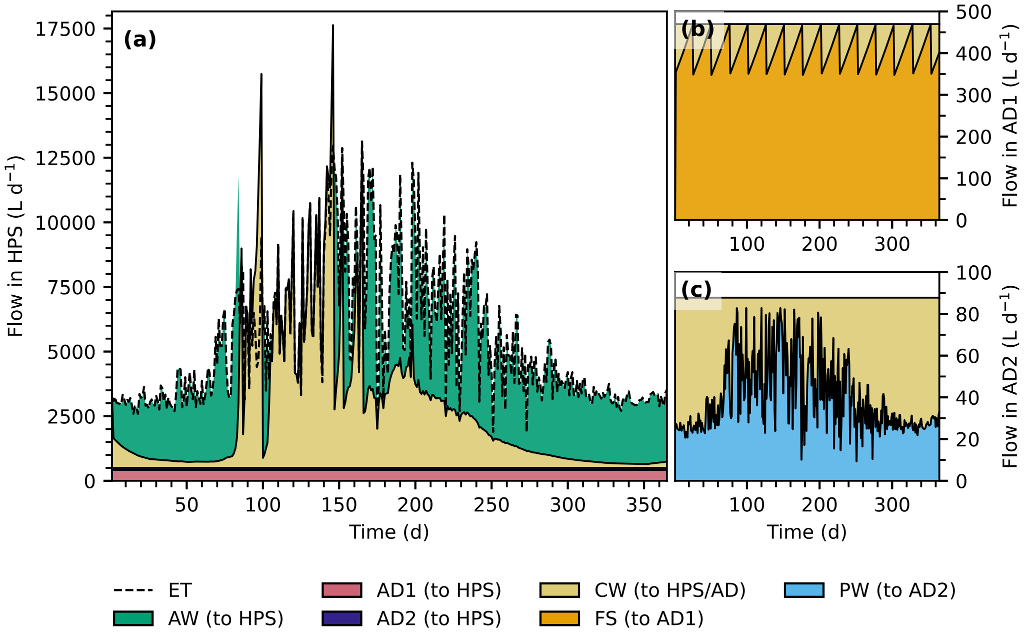

Figure S4 water flows of the integrated aqua-agriculture network in Amsterdam, related to Figure 3. Subplot (a) shows the water flows towards the HPS from two anaerobic digesters, one digesting fish sludge (AD1) and the other plant waste (AD2), and nutrient-rich water from the recirculating aquaculture system (AW). Subplots (b) and (c) show the incoming anaerobic digester flows consisting of sludge water (SW), plant waste (PW), and additional clean water (CW1, CW2). In this case, the flows do not always align with the greenhouse evapotranspiration (ET), denoted by the black dashed-dotted line. The solver chooses to discharge water from the hydroponic system as can be derived from the extra bursts of clean water (CW). This allows the RAS to continue with water transfer to the HPS because dumping water from the RAS has a higher price due to having a higher nutrient concentration as can be seen from Figure S3 subplot (a).

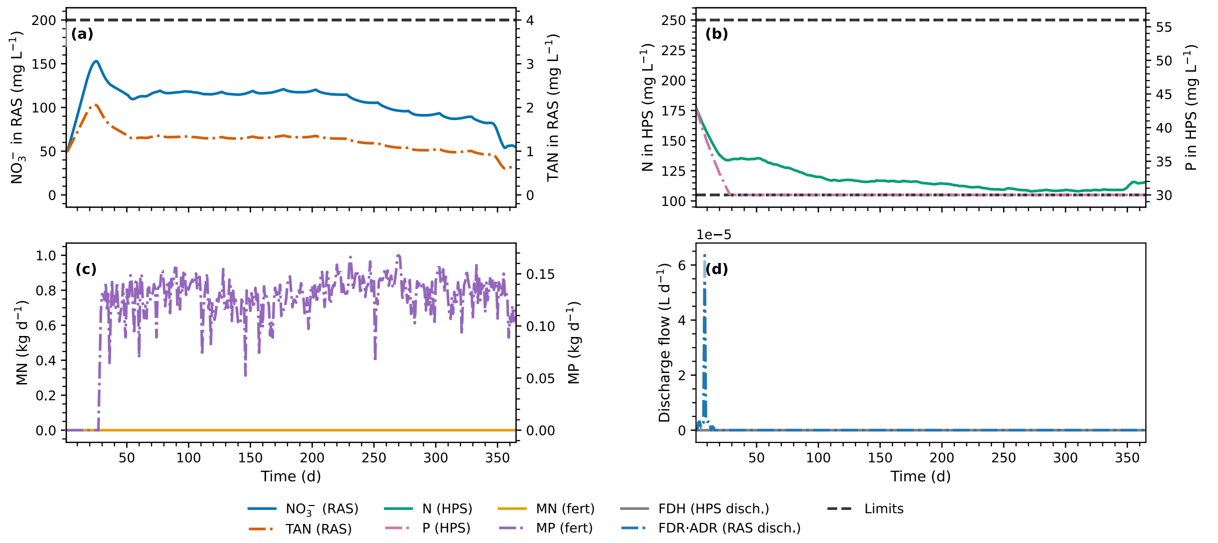

Figure S5 RAS and HPS nutrient and discharge profiles in a decoupled aquaponics network in Jakarta, related to Table 1. Nutrient and discharge profiles in a decoupled aquaponics network in Jakarta with an optimal HPS cultivation area of 1800 m^2. P becomes limiting and requires supplementation as can be observed from subplot (c). No discharge is required.

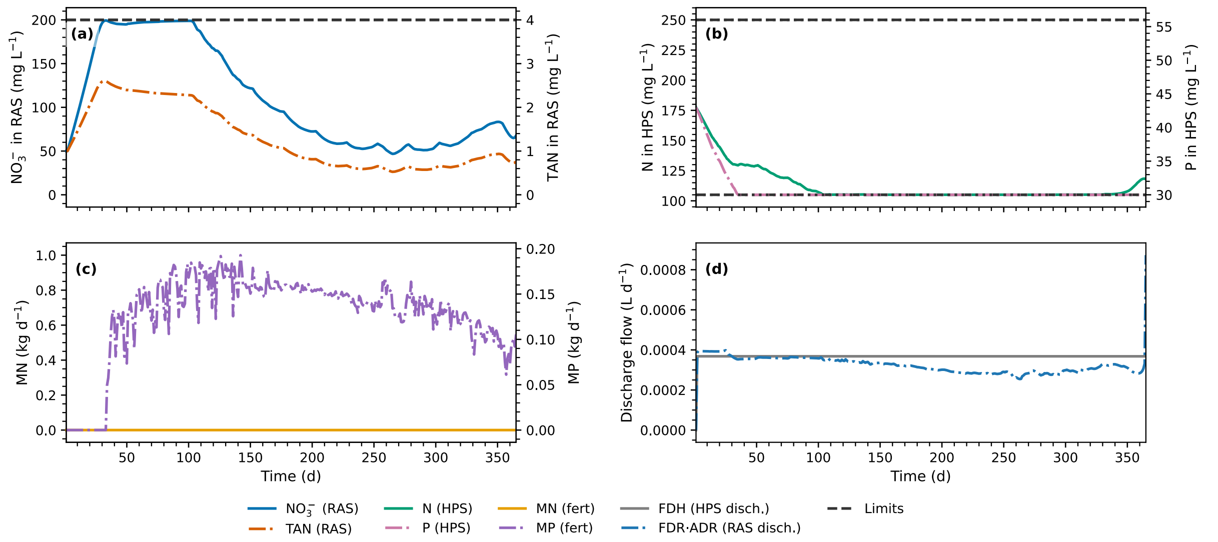

Figure S6 RAS and HPS nutrient and discharge profiles in a decoupled aquaponics network in Cairo, related to Table 1. Nutrient and discharge profiles in a decoupled aquaponics network in Cairo with an optimal HPS cultivation area of 1575 m^2. P becomes limiting and requires supplementation as can be observed from subplot (c). No discharge is required.

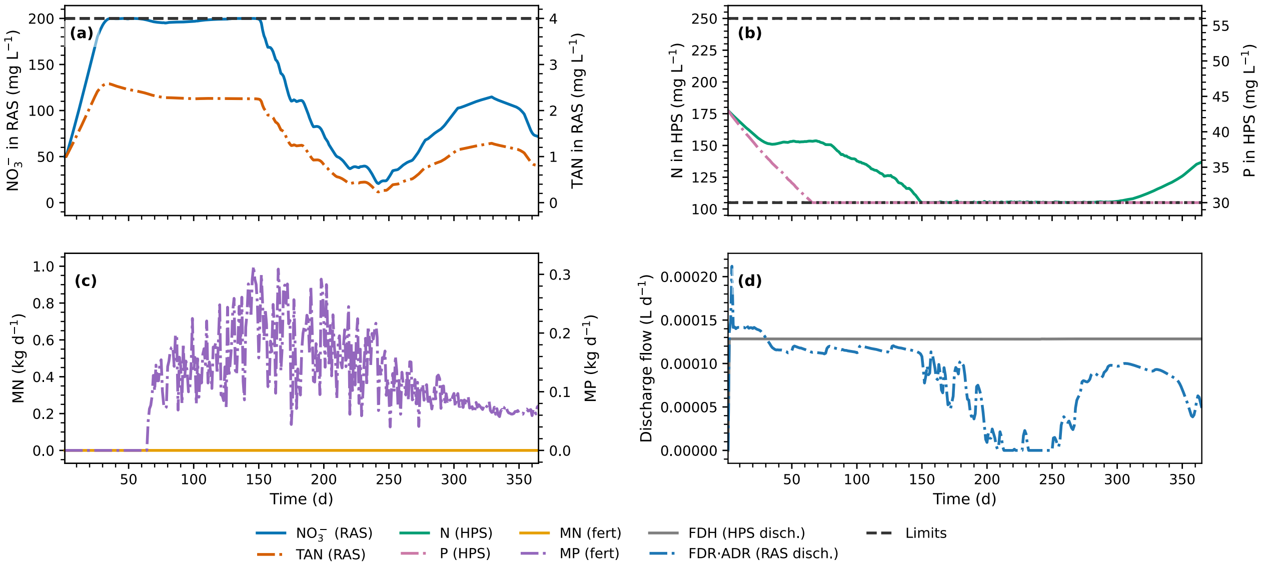

Figure S7: RAS and HPS nutrient and discharge profiles in a decoupled aquaponics network in Amsterdam, related to Table 1. Nutrient and discharge profiles in Amsterdam with an optimal HPS cultivation area of 2250 m^2. P becomes limiting and requires supplementation as can be observed from subplot (c). No discharge is required.

Supplemental tables

Table S1: Parameters used to describe RAS components.

Parameter	Description	Value	Unit	Reference
Fish species	Nile Tilapia		-	S[1]
T_{RAS}	Water temperature RAS.	29	$°C$	
Tanks	Number of fish tanks.	10	-	S[2] S[3]
V_{RAS}	Total volume of tanks.	100	m^3	
$n_{Average}^{fish}$	Fish per unit of volume.	130	$fish/m^3$	
Fingerling weight	Start weight fish.	3	$gram$	
Harvest weight	Maximum weight fish.	600	$gram$	
ρ_{max}	Maximum fish stocking density.	80	$\frac{kg}{m^3}$	
Growth cycle	Days till maximum weight is achieved.	252	$days$	
DM_{feed}	Dry matter content in fish feed	90%	$\frac{kg_{DM}}{kg_{feed}}$	
w_{feed}^{N}	Weight fraction of N in feed	5%	$\frac{kg_N}{kg_{feed}}$	
w_{feed}^{P}	Weight fraction of P in feed.	1%	$\frac{kg_P}{kg_{feed}}$	
w_{feed}^{COD}	COD as a result of the feed added.	1.4	$\frac{kg_{COD}}{kg_{feed}}$	
δ	Fraction of feed ingested by fish.	82%	-	
ε_{faeces}^{N}	Average fraction of N eaten by fish that is excreted as feces by fish.	11%	$\frac{kg_N}{kg_{feed}}$	S[4]
ε_{faeces}^{P}	Average fraction of P eaten by fish that is excreted as feces by fish.	45%	$\frac{kg_P}{kg_{feed}}$	
$\varepsilon_{soluble}^{TAN}$	Fraction of N eaten by fish that is excreted as soluble TAN by fish	44%	$\frac{kg_N}{kg_{feed}}$	

$\varepsilon_{soluble}^{P}$	Fraction of P eaten by fish that is excreted as soluble P	3.5%	$\frac{kg_P}{kg_{feed}}$	
TSS_{sludge}	Weight fraction of the TSS in the sludge.	0.4-3.0% Model setting: 2%	-	S[5-8]
DM_{TSS}	Fraction of feed DM that ends up as TSS in the sludge.	25%	$\frac{kg_{TSS}}{kg_{DM}}$	S[9]
η_{bf}^{RAS}	Efficiency of the nitrification process in the biofilter	99%	-	S[1]
η_{den}^{RAS}	Loss of nitrate due to uncontrolled denitrification process	Average 10%	-	S[10]

Table S2: Parameters used to describe AD components.

Parameter	Description	Value	Unit	Reference
$\eta_{N}^{AD_1}$	Fraction of N in solid sludge that is mineralized to TAN.	86%	-	S[10], S[11]
$\eta_{P}^{AD_1}$	Fraction of P in solid sludge that is mineralized to soluble P.	58%	-	
$\eta_{N}^{AD_2}$	Fraction of N in plant waste that is mineralized to TAN.	85%	-	S[12]
$\eta_{P}^{AD_2}$	Fraction of P in plant waste that is mineralized to soluble P.	75%	-	
ϕ_{waste}^{crop}	Fraction of lettuce mass being wasted.	20%	$\frac{kg_{Waste}}{kg_{Lettuce}}$	
DM_{Crop}	Dry matter content lettuce.	6%	$\frac{kg_{DM}}{kg_{lettuce}}$	
VS_{crop}	VS content lettuce	4.58	$\frac{kg_{VS}}{kg_{lettuce}}$	
ϕ_{DM}^{N}	Fraction of N being part of crop dry matter.	5.6%	$\frac{kgN}{kgDM}$	S[13]
η_{bf}	Efficiency of the nitrification process in the biofilter post anaerobic digestion.	99%	-	S[1]
η_{den}	Efficiency of the denitrification process during anaerobic digestion.	99%	-	Assumed
$\eta_{COD}^{AD_1}$	COD content in fish sludge that is converted during anaerobic digestion. Same assumed for manure as in commercial reactors 80-90% conversion can be achieved (Lier et al., 2008).	90%	-	S[14-15]
$\eta_{COD}^{AD_2}$	COD content in lettuce waste that is converted during anaerobic digestion	86%	-	S[12]
Φ_{COD}^{crop}	Waste COD fraction of plant DM	1.2	$\frac{kgCOD}{kgDM}$	S[16]
$\gamma_{biogas}^{AD_1}$	Biogas yield per unit of COD fish sludge. Assumed for manure similar yield.	0.24-0.33; Model setting=0.33	$\frac{biogas\ m^3}{kg_{COD}}$	S[17-18]

$\phi_{methane}^{AD_1}$	Fraction of methane in biogas obtained from manure and or fish sludge.	53%	$\frac{m^3 CH_4}{m_3\ biogas}$	S[14]
$Y_{biogas}^{AD_2}$	Biogas yield per unit of COD lettuce waste	0.540	$\frac{biogas\ m^3}{kg_{COD}}$	S[12]
$\phi_{methane}^{AD_2}$	Fraction of methane in biogas	59%	$\frac{m^3 CH_4}{m_3\ biogas}$	
$\Phi_{digestate}$	Fraction of water remaining in the digestate	10%	-	Assumed
V_{OLR}	Optimal VS loading rate per unit volume of reactor.	1.5	$\frac{g}{L}$	S[19]
$VS(t)$	Volatile solid target level in the reactor treating plant waste.	allowed to range between 1-2	$\frac{g}{L}$	
TS_{manure}	Total solids in diluted manure.	53	$\frac{g}{L}$	S[20]
$VS_{chicken}$<br>VS_{pig}<br>VS_{cattle}	Total volatile solids in diluted manure.	44.7<br>40.0<br>33.8	$\frac{g}{L}$	
$TAN_{chicken}$<br>TAN_{pig}<br>TAN_{cattle}	Total ammonia nitrogen in diluted manure	0.330<br>0.364<br>0.255	$\frac{g}{L}$	
$P_{chicken}^{solids}$<br>P_{pig}^{solids}<br>P_{cattle}^{solids}	Solid P in diluted manure.	0.783<br>0.585<br>0.288	$\frac{g}{L}$	
$N_{chicken}^{solids}$<br>N_{pig}^{solids}<br>N_{cattle}^{solids}	Solid N in diluted manure.	2.028<br>1.054<br>1.106	$\frac{g}{L}$	
COD_{manure}^{Total}	Total COD content in manure with a total solid content of 10%.	103	$\frac{g}{L}$	S[21]
COD_{manure}^{solids}	Solid COD content in manure diluted to 5% TS content.	25	$\frac{g}{L}$	
$COD_{manure}^{soluble}$	Soluble COD content in manure diluted to 5% TS content.	25	$\frac{g}{L}$	
HRT_1	Hydraulic retention time of AD1	30	Days	S[6]
HRT_2	Hydraulic retention time of AD2	25	Days	S[12]
$T_{reactors}$	Reactor operating temperature	40	°C	S[15]
$T_{outside}$	Outside temperature in Jakarta and Cairo		°C	S[22]
U_{tot}	Estimated total heat transfer coefficients reactor after estimating reactor dimensions for given height of 4 meter and from top, bottom, and lateral given coefficients.	0.95<br>0.6<br>0.6	$\frac{W}{m^2 \cdot °C}$	S[23]
CV_m	Caloric energy of methane	9.7	$\frac{kWh}{m^3}$	
E	Efficiency of the methane energy yield	0.90	-	S[24]
C_p	Specific heat capacity waste water	4182	$\frac{J}{kg \cdot °C}$	S[25]
ρ	Density waste water	1000	$\frac{kg}{m^3}$	Assumed

Table S.3: Parameters used to describe HPS components.

Parameter	Description	Value	Unit	Reference
A_{HPS}	Area of the the hydroponic greenhouse in Jakarta and Cairo.	Optimized; decision variable.	m^2	

	Note this is not the planting area.			
H_{HPS}	Height of hydroponics in deep water culture.	0.2	m	Chosen based on conventional deep water culture.
a_{crops}	Fraction of hydroponic greenhouse area that is cropped.	0.9	-	S[2]

Method S1:System model equations and descriptions.

The aquaculture recirculating system

The mass balance equations below describe the aquaculture water flow and nutrient concentrations, which were determined by the outgoing fluxes and the total nutrients excreted by the fish.

The volume of the recirculating aquaculture system was remained constant, that is the time derivative, denoted by the dot: $\dot{V}_{RAS} = 0$. This is realized by replacing the outgoing discharge F_{DC}^{RAS} and sludge water F_{SW}^{RAS} fluxes with clean fresh water F_{in}^{RAS}.

$$\dot{V}_{RAS} = F_{in}^{RAS} - F_{SW}^{RAS} - F_{DC}^{RAS} \quad \textit{(Eq. S.1)}$$

$$F_{SW}^{RAS} = M_{feed} \cdot \phi_{DM} \cdot \phi_{TSS} \cdot \frac{(1-\phi_{TSS}^{SW})}{\phi_{TSS}^{SW}} \quad \textit{(Eq. S.2)}$$

The sludge water is determined by the fraction of dry matter feed (ϕ_{DM}) ending up as total suspended solids (ϕ_{TSS}) and forming the weight fraction of fish sludge (ϕ_{TSS}^{SW}). The aquaculture soluble and solid phosphorus (P) concentrations are determined by the incoming feed mass M_{feed}, fish excretion constants and outgoing fluxes. Here ϕ_p is the weight fraction of P in the feed, δ_{feed} is the fraction of eaten feed, ε_P and $\varepsilon_{Pfaeces}$ are the fraction of soluble and solid excreted P. It is assumed that all solid particles are mechanically collected and end up as fish sludge F_{SW}.

$$\dot{C}_{P_{soluble}}^{RAS} = \frac{M_{feed} \cdot \phi_p \cdot \delta_{feed} \cdot \varepsilon_P - (F_{SW} + F_{DC}^{RAS}) \cdot C_{P_{soluble}}^{RAS}}{V_{RAS}} \quad \textit{(Eq. S.3)}$$

$$\dot{C}_{P_{solid}}^{RAS} = \frac{M_{feed} \cdot \phi_p \cdot (1 - \delta_{feed} + \delta_{feed} \cdot \varepsilon_{Pfaeces}) - F_{SW}^{RAS} \cdot C_{P_{solid}}^{RAS}}{V_{RAS}} \quad \textit{(Eq. S.4)}$$

Likewise, total ammonia nitrogen (TAN), nitrate (NO_3^-), and solid nitrogen (N) particle concentration dynamics are described. Most TAN is converted to NO_3^- by a nitrification constant (η_{bf}).

$$\dot{C}_{TAN}^{RAS} = \frac{M_{feed} \cdot \phi_N \cdot \delta_{feed} \cdot \varepsilon_{TAN} \cdot (1 - \eta_{bf}) - (F_{SW}^{RAS} + F_{DC}^{RAS}) \cdot C_{TAN}^{RAS}}{V_{RAS}} \quad \textit{(Eq. S.5)}$$

$$\dot{C}_{NO_3}^{RAS} = \frac{M_{feed} \cdot \phi_N \cdot \delta_{feed} \cdot \varepsilon_{TAN} \cdot \eta_{bf}^{RAS} - (F_{SW}^{RAS} + F_{DC}^{RAS}) \cdot C_{NO_3}^{RAS}}{V_{RAS}} \quad \text{(Eq. S.6)}$$

$$\dot{C}_{N_{solid}}^{RAS} = \frac{M_{feed} \cdot \phi_N \cdot (1 - \delta_{feed} + \delta_{feed} \cdot \delta N_{faeces}) - F_{SW}^{RAS} \cdot C_{N_{solid}}^{RAS}}{V_{RAS}} \quad \text{(Eq. S.7)}$$

We also described the potential soluble and solid chemical oxygen demand concentrations originating from the fish feed to determine potential biogas yield. Here γ_{COD} is the COD yield per unit of fish feed.

$$\dot{C}_{COD_{soluble}}^{RAS} = \frac{M_{feed} \cdot \gamma_{COD} \cdot \delta_{feed} \cdot \varepsilon_{COD} - (F_{SW} + F_{DC}^{RAS}) \cdot C_{COD_{soluble}}^{RAS}}{V_{RAS}} \quad \text{(Eq. S.8)}$$

$$\dot{C}_{COD_{solid}}^{RAS} = \frac{M_{feed} \cdot \gamma_{COD} \cdot (1 - \delta_{feed} + \delta_{feed} \cdot \delta COD_{faeces}) - F_{SW} \cdot C_{COD_{solid}}^{RAS}}{V_{RAS}} \quad \text{(Eq. S.9)}$$

The anaerobic digestion system

$$\dot{V}_{AD_1} = F_{in}^{AD1} + F_{SW}^{RAS} + F_{CM}^{AD1} - F_{out}^{AD_1} \quad \text{(Eq. S.10)}$$

$$F_{out}^{AD1} = F_{SW}^{max} + F_{CM_{max}}^{AD1} \quad \text{(Eq. S.11)}$$

$$F_{in}^{AD1} = F_{SW}^{max} + F_{CM_{max}}^{AD1} - F_{SW} - F_{CM}^{AD1} \quad \text{(Eq. S.12)}$$

$$V_{AD1} = F_{out}^{AD1} \cdot HRT_1 \quad \text{(Eq. S.13)}$$

$$\dot{V}_{AD_2} = F_{in}^{AD2} + F_{ET} \cdot C_N^{HPS} \cdot \phi_{waste}^{crop} \cdot \left(\frac{1}{\phi_{DM}^{N}}\right) \cdot \frac{(1 - DM_{crop})}{DM_{crop}} + F_{CM}^{AD2} - F_{out}^{AD2} \quad \text{(Eq. S.14)}$$

$$F_{out}^{AD2} = \left(\frac{V_{AD2}}{HRT_2}\right) \quad \text{(Eq. S.15)}$$

$$F_{in}^{AD2} = F_{out}^{AD2} - F_{ET} \cdot C_N^{HPS} \cdot \phi_{waste}^{crop} \cdot \left(\frac{1}{\phi_{DM}^{N}}\right) \cdot \frac{(1 - DM_{crop})}{DM_{crop}} - F_{CM}^{AD2} \quad \text{(Eq. S.16)}$$

$V_{AD2} = \frac{VSmax|average}{VOLR}$ *(see eq. S.32-36)* *(Eq. S.17)*

The phosphorus and nitrogen concentrations in the reactors are determined by the amount of phosphorus and nitrogen in the incoming fish sludge (F_{SW}^{RAS}), manure (F_{Manure}) , and crop nutrient uptake ending up in inedible parts ($F_{ET}^{max} \cdot C_{Nutrient}^{HPS} \cdot \phi_{waste}^{crop}$). The mass balances describing the rate of change for both soluble and solid nutrient concentrations is given below.

$$\dot{C}_{P_{soluble}}^{AD_1} = \frac{F_{SW}^{RAS} \cdot (C_{P_{soluble}}^{RAS} + C_{P_{solid}}^{RAS} \cdot \eta_P^{AD_1}) + F_{manure}^{AD1} \cdot (C_{P_{solid}}^{CM} \cdot \eta_P^{AD_1}) - F_{out}^{AD_1} \cdot C_P^{AD_1}}{V_{AD_1}} \quad \text{(Eq. S.18)}$$

$$\dot{C}_{TAN}^{AD_1} = \frac{F_{SW}^{RAS} \cdot (C_{TAN}^{RAS} + C_{N_{solid}}^{RAS} \cdot \eta_N^{AD_1}) + F_{manure}^{AD1} \cdot (C_{TAN}^{CM} + C_{N_{solid}}^{CM} \cdot \eta_N^{AD_1}) - F_{out}^{AD_1} \cdot C_{TAN}^{AD_1}}{V_{AD_1}} \quad \text{(Eq. S.19)}$$

$$\dot{C}_{P_{soluble}}^{AD_2} = \frac{F_{manure}^{AD2} (C_{P_{solid}}^{CM} \cdot \eta_P^{AD_1}) + C_P^{HPS} \cdot F_{ET} \cdot \phi_{waste}^{crop} \cdot \eta_P^{AD_2} - F_{out}^{AD_2} \cdot C_P^{AD_2}}{V_{AD_2}} \quad \text{(Eq. S.20)}$$

$$\dot{C}_{TAN}^{AD_2} = \frac{F_{manure}^{AD2} \cdot \left(C_{TAN}^{CM} + C_{N_{solid}}^{CM} \cdot \eta_N^{AD_1}\right) + F_{ET} \cdot C_N^{HPS} \cdot \phi_{waste}^{crop} \cdot \eta_N^{AD_2} - F_{out}^{AD_2} \cdot C_{TAN}^{AD_2}}{V_{AD_2}} \quad \text{(Eq. S.21)}$$

Nitrate content in the fish sludge was assumed to become completely denitrified.

$$M_{N_2} = F_{SW}^{RAS} \cdot C_{NO_3}^{RAS} \quad \text{(Eq. S.22)}$$

Note that the flow of manure was only applied in the scenario of operating an industrial aquaponics network in Cairo Egypt and Amsterdam, the Netherlands. The manure in our study was diluted with a TS level similar to fish sludge ranging between 1.5-2.5% because high TS and ammonia levels can be difficult to anaerobically digest. The chemical oxygen demand (COD) balances across the anaerobic digesters are included as well to estimate the biogas production. The COD conversion efficiency is denoted by $\eta_{TSS}^{AD_1}$ and $\eta_{PW}^{AD_2}$.

$$\dot{C}_{COD}^{AD_1} = \frac{F_{SW}^{RAS} \cdot \left(C_{COD_{soluble}}^{RAS} + C_{COD_{solid}}^{RAS} \cdot \eta_{COD}^{AD_1}\right) + F_{manure}^{AD1} \cdot \left(C_{COD_{soluble}}^{CM} + C_{COD}^{CM} \cdot \eta_{COD}^{AD_1}\right) - F_{out}^{AD_1} \cdot C_{COD}^{AD_1}}{V_{AD_1}} \quad \text{(Eq. S.23)}$$

$$\dot{C}_{COD}^{AD_2} = \frac{F_{ET} \cdot C_N^{HPS} \cdot \phi_{waste}^{crop} \cdot \left(\frac{1}{\phi_{DM}^{N}}\right) \cdot \phi_{COD}^{crop} \cdot \eta_{COD}^{AD_2} - F_{out}^{AD_2} \cdot C_{COD}^{AD_2} + F_{manure}^{AD2} \cdot \left(C_{COD_{soluble}}^{CM} + C_{COD}^{CM} \cdot \eta_{COD}^{AD_1}\right)}{V_{AD_2}} \quad \text{(Eq. S.24)}$$

An energy balance across the reactors was defined to estimate the net energy yield from methane. Net energy was obtained after determining required energy to heat the incoming flows and maintain reactor temperature due to dissipation of heat. Here the C_{vm} is the volumetric caloric energy yield from methane in kWh m^{-3}, E is the efficiency of methane energy yield, γ is the biogas yield from the soluble COD, $\phi_{methane}$ are the methane yields, ρ is the density of water in kg m^{-3}, C_p is the specific heat capacity of water in kJ kg^{-1} °C^{-1},U_{tot} is the total heat transfer coefficient of each reactor in $w\, m^{-2}\, °C^{-1}$, T is the target temperature of the digesters in °C, and $T_{outside}$ is the outside temperature.

$$E_{net} = \sum E_{biogas} - \sum E_{heat} - \sum E_{dissipation} \quad \text{(Eq. S.25)}$$

$$E_{biogas}^{AD_1} = F_{out}^{AD_1} \cdot C_{COD}^{AD_1} \cdot \gamma_{biogas}^{AD_1} \cdot \phi_{methane}^{AD_1} \cdot C_{vm} \cdot E \quad \text{(Eq. S.26)}$$

$$E_{biogas}^{AD_2} = F_{out}^{AD_2} \cdot C_{COD}^{AD_2} \cdot \gamma_{biogas}^{AD_2} \cdot \phi_{methane}^{AD_2} \cdot C_{vm} \cdot E \quad \text{(Eq. S.27)}$$

$$\dot{E}_{heat}^{AD_1} = F_{in}^{AD_1} \cdot C_p \cdot \rho \cdot \left(T_{AD_1} - T_{Outside}\right) \quad \text{(Eq. S.28)}$$

$$\dot{E}_{heat}^{AD_2} = F_{in}^{AD_1} \cdot C_p \cdot \rho \cdot \left(T_{AD_2} - T_{Outside}\right) \quad \text{(Eq. S.29)}$$

$$\dot{E}_{Dissipation}^{AD_1} = U_{tot}^{AD_1} \cdot (T_{AD_1} - T_{Outside}) \quad \text{(Eq. S.30)}$$

$$\dot{E}_{Dissipation}^{AD_2} = U_{tot}^{AD_2} (T_{AD_2} - T_{Outside}) \quad \text{(Eq. S.31)}$$

The plant-waste digester was designed based on the volatile solids (VS) content of the waste stream, with manure added when VS levels were insufficient or additional nutrients were

needed in the HPS. In Cairo, the maximum potential VS loading from plant waste was initially determined by the highest nitrogen concentration in the HPS and the maximum outgoing evapotranspiration flux, as defined in Equation S.32. The digester volume was then sized according to Equation S.33 to maintain an optimal volatile solid organic loading rate (VOLR) of 1.5 g VS L^{-1} d^{-1} when the maximum amount of plant waste was processed. The actual VOLR is often lower since biomass yields are rarely at maximum. To maintain levels close to the optimal VOLR, manure is added to the digester.

In Jakarta, a different approach was taken, omitting manure due to the region's relatively stable climate, which led to more consistent crop yields. A soft constraint was applied to keep the average VOLR near the optimal 1.5 g VS L^{-1} d^{-1}. This approach ensured that occasional cloudy or cooler days with lower harvests did not cause constraint violations.

$$VS_{Max}^{Plant} = F_{ET}^{max} CN_{max}^{HPS} \Phi_{Waste}^{Crop} \left(\frac{1}{\phi_{DM}^{N}}\right)\left(1 + \frac{(1-DM_{crop})}{DM_{crop}}\right) VS_{crop} \quad \text{(Eq. S.32)}$$

$$V_{AD2}^{CAI,AMS} = \frac{VS_{Max}^{Plant} + VS_{Max}^{Manure}}{VOLR} \quad \text{(Eq. S.33)}$$

$$VS_{in} = \frac{\left(F_{ET}\left(C_{NO_3}^{HPS} + C_{TAN}^{HPS}\right)\Phi_{Waste}^{Crop}\left(\frac{1}{\phi_{DM}^{N}}\right)\left(1+\frac{(1-DM_{crop})}{DM_{crop}}\right) VS_{crop} + F_{manure}^{AD2} VS_{manure}\right)}{V_{AD2}} \quad \text{(Eq. S.34)}$$

$$V_{AD2}^{Jakarta} = \frac{\left(F_{ET}^{Average} CN_{Average}^{HPS} \Phi_{Waste}^{Crop}\left(\frac{1}{\phi_{DM}^{N}}\right)\left(1+\frac{(1-DM_{crop})}{DM_{crop}}\right) VS_{crop}\right)}{VOLR} \quad \text{(Eq. S.35)}$$

$$VS_{avg} = \frac{\int_0^T VS_{in}(t)}{T} \quad \text{(Eq. S.36)}$$

The hydroponic greenhouse and climate model

The greenhouse climate model is used as described in previous work[1]. The model uses a staggered lettuce production scenario of 18 lettuce per m^2 having an initial and maximum leaf area index of 6.5 cm^2 m^{-2} and 3 m^2 m^{-2} with a growth cycle of 50 days to maintain an average leaf area index of 1.5 and subsequently uses hourly humidity, radiation, and temperature inputs from Jakarta and Cairo to calculate daily lettuce transpiration rates (L m^{-2} d^{-1}). The mass balances equations below describe the nutrient dynamics in the hydroponic greenhouse driven by the total crop evapotranspiration flux (F_{EV}).

$$\dot{V}_{HPS} = F_{in}^{HPS} + \left(F_{out}^{AD_1} + F_{out}^{AD_2}\right) \cdot \left(1 - \Phi_{digestate}\right) + F_{DC}^{RAS} \cdot (1 - \alpha_{DC}) - F_{ET} - F_{DC}^{HPS} \quad \text{(Eq. S.37)}$$

With, $F_{ET} = A_{HPS} \cdot \alpha_{crops} \cdot ET_c$

$$\dot{C}_P^{HPS} = (F_{out}^{AD_1} \cdot C_{P_{soluble}}^{AD_1} + F_{out}^{AD_2} \cdot C_{P_{soluble}}^{AD_2}) \cdot (1 - \Phi_{digestate}) + F_{DC}^{RAS} \cdot \alpha_{DC} \cdot C_{P_{soluble}}^{RAS} + M_P - (F_{ET} + F_{DC}^{HPS}) \cdot C_P^{HPS} \quad \text{(Eq. S.38)}$$

$$\dot{C}_{TAN}^{HPS} = (F_{out}^{AD_1} \cdot C_{TAN}^{AD_1} + F_{out}^{AD_2} \cdot C_{TAN}^{AD_2}) \cdot (1 - \Phi_{digestate}) \cdot (\alpha_{bf} \cdot (1 - \eta_{bf}) + (1 - \alpha_{bf})) + F_{DC}^{RAS} \cdot \alpha_{DC} \cdot C_{TAN}^{RAS} + M_{TAN} - (F_{ET} + F_{DC}^{HPS}) \cdot C_{TAN}^{HPS} \quad \text{(Eq. S.39)}$$

$$\dot{C}_{NO_3}^{HPS} = (F_{out}^{AD_1} \cdot C_{TAN}^{AD_1} + F_{out}^{AD_2} \cdot C_{TAN}^{AD_2}) \cdot (1 - \Phi_{digestate}) \cdot \alpha_{bf} \cdot \eta_{bf} + F_{DC}^{RAS} \cdot \alpha_{DC} \cdot C_{NO_3}^{RAS} + M_{NO_3} - (F_{ET} + F_{DC}^{HPS}) \cdot C_{NO_3}^{HPS} \quad \text{(Eq. S.40)}$$

The volume of the hydroponic greenhouse remained constant by replacing the outgoing evapotranspiration and discharge F_{DC}^{HPS} with fluxes from the aquaculture unit $F_{DC}^{RAS} \cdot \alpha_{DC}$, anaerobic digesters F_{out}^{AD}, and clean fresh water F_{in}^{HPS}. Part of the anaerobic digestion effluent ends up in the digestate, which is denoted by $\Phi_{digestate}$. In case nutrients drop below the target concentrations, synthetic fertilizer is added denoted by M_P, M_{NO_3}, M_{TAN}. Furthermore, part of the aquaculture and anaerobic digestion effluents pass a nitrification unit that turn ammonia into nitrates as denoted by the α_{bf}, which is the fractional flow sent to the biofilter and the conversion efficiency denoted by η_{bf}.

Method S2: Python optimization backbone code

```
import numpy as np
from gekko import GEKKO
import pandas as pd
# ----------------------------
# Constants
# ----------------------------
const = {
    'AHPS': 5700,
    'Ac': 0.9,
    'DM': 0.9,
    'TSS': 0.25,
    'sTSS': 0.02,
    'wP': 0.01,
    'wN': 0.05,
    'dF': 0.82,
    'ePF': 0.45,
    'ePS': 0.035,
    'iP': 0.58,
    'eTAN': 0.44,
    'eNF': 0.11,
    'bf': 0.99,
```

```
'denRAS': 0.10,
'iN': 0.86,
'denAD': 0.99,
'DMplant': 0.06,
'drm_N': 0.056,
'drm_P': 0.0084,
'Pwaste': 0.20,
'pw_iP': 0.75,
'pw_iN': 0.847,
'denHPS': 0.1,
'Vras': 100 * 1000,  # L
'HRT': 25,
'sf': 1e-3,
'HRT_pw': 25,
'wCOD': 1.4,
'eCOD': 0.53,
'eCODF': 0.14,
'iCOD': 0.98,
'wCODplant': 1.2,
'iTSS': 0.90,
'Ybg': 0.24,
'rho': 1000,
'hc': 4.182,
'E': 0.9,
'CVm': 9.7,
'Tad': 40,
'Hr': 4,
'Utop': 0.95,
'U': 0.6,
'f_meth': 0.53,
'iCODpw': 0.86,
'f2_meth': 0.59,
'Ybg2': 0.54,
'VOLR': 1.4,
'VS': 0.039 * (6 / 5.1),
'VS_CM': 35,
'TS_CM': 50,
'TS_FS': 20,
'CM_P': 1045e-3,
'CM_N': 2200e-3,
'CM_TAN': 598e-3,
```

```
    'CowN': 1106e-3,
    'CowTAN': 255e-3,
    'CowP': 288e-3,
    'CowVS': 33.83,
    'PigP': 585e-3,
    'PigN': 1054e-3,
    'PigVS': 40.04,
    'PigTAN': 364e-3,
    'ChickN': 2028e-3,
    'ChickTAN': 329e-3,
    'ChickP': 783e-3,
    'ChickVS': 44.70
}
# -----------------------------
# Required external helpers
# -----------------------------
def generate_feed_series(fish_overrides=None):
    raise NotImplementedError("Provide generate_feed_series(...)")


def build_timeseries_dataframe(*args, **kwargs):
    raise NotImplementedError("Provide build_timeseries_dataframe(...)")


def compute_kpis(df, const_local):
    raise NotImplementedError("Provide compute_kpis(...)")




def run_ocp(
    data: pd.DataFrame,
    const_overrides=None,
    fish_overrides=None,
    climate_col: str = 'Jakarta',
    A_HPS: float = 1750,
    LB=(30, 56),
    UB=(105, 250),
    FCMmax: float = 1000,
    FCM2max: float = 800,
    remote: bool = True,
    disp: bool = True
):
    """
```

```
Dynamic OCP (GEKKO, IMODE=6) with manure type competition and objective as a functional.

data must include:
  - 'Day'
  - climate_col (ET trajectory, e.g. L/m2/day)
"""

# -----------------------------
# 1) Constants
# -----------------------------
c = const.copy()
if const_overrides:
    c.update(const_overrides)
if A_HPS is not None:
    c['AHPS'] = float(A_HPS)
AHPS = c['AHPS']

manure = {
    'cow':   {'P': c['CowP'],   'Norg': c['CowN'],   'TAN': c['CowTAN'],   'VS': c['CowVS'],   'TS': 50.0},
    'pig':   {'P': c['PigP'],   'Norg': c['PigN'],   'TAN': c['PigTAN'],   'VS': c['PigVS'],   'TS': 50.0},
    'chick': {'P': c['ChickP'], 'Norg': c['ChickN'], 'TAN': c['ChickTAN'], 'VS': c['ChickVS'], 'TS': 50.0},
}

# -----------------------------
# 2) Disturbances (align lengths)
# -----------------------------
t_full = data['Day'].values[1:]
ET_full = data[climate_col].values[1:]  # L/m2/day

Feed_series = generate_feed_series(fish_overrides=fish_overrides)  # kg/day

n_days = min(len(t_full), len(ET_full), len(Feed_series))
t = t_full[:n_days]
Es = ET_full[:n_days]
Fs = np.asarray(Feed_series[:n_days], dtype=float)

# GEKKO time grid
ts = np.linspace(float(t[0]), float(t[-1]), n_days)

# -----------------------------
# 3) Initial conditions
```

```
# -----------------------------
sf = c['sf']
x0 = [
    2.5 * sf, 485.32 * sf, 50 * sf, 1.0 * sf, 1254.81 * sf,
    253 * sf, 720 * sf, 0.0, (LB[1] + UB[1]) * 0.5 * sf, (LB[0] + UB[0]) * 0.5 * sf, 0 * sf
]

# -----------------------------
# 4) Build GEKKO model
# -----------------------------
m = GEKKO(remote=remote)
m.time = ts

# Params
F = m.Param(value=Fs)     # kg/day
ET = m.Param(value=Es)    # L/m2/day

FCM_max = float(FCMmax)
FCM2_max = float(FCM2max)

# -----------------------------
# 5) Decision variables (MVs)
# -----------------------------
# core controls
FDR  = m.MV(value=0, lb=0, ub=100)   # m3/day (?) (you later *1000)
FDH  = m.MV(value=0, lb=0, ub=100)
MP   = m.MV(value=0, lb=0, ub=100)
MNO3 = m.MV(value=0, lb=0, ub=100)
MTAN = m.MV(value=0, lb=0, ub=100)
ADR  = m.MV(value=1.0, lb=0, ub=1)
BF   = m.MV(value=1.0, lb=1, ub=1)   # fixed

# manure MVs (AD1)
FCM_cow   = m.MV(value=0, lb=0, ub=FCM_max)
FCM_pig   = m.MV(value=0, lb=0, ub=FCM_max)
FCM_chick = m.MV(value=0, lb=0, ub=FCM_max)
FCM1_tot  = m.Intermediate(FCM_cow + FCM_pig + FCM_chick)
m.Equation(FCM1_tot <= FCM_max)

# manure MVs (AD2)
FCM2_cow   = m.MV(value=0, lb=0, ub=FCM2_max)
```

```
    FCM2_pig   = m.MV(value=0, lb=0, ub=FCM2_max)
    FCM2_chick = m.MV(value=0, lb=0, ub=FCM2_max)
    FCM2_tot   = m.Intermediate(FCM2_cow + FCM2_pig + FCM2_chick)
    m.Equation(FCM2_tot <= FCM2_max)

    # Activate MVs + (optional) smoothing on manure
    for mv in [FDR, FDH, MP, MNO3, MTAN, ADR, BF,
               FCM_cow, FCM_pig, FCM_chick, FCM2_cow, FCM2_pig, FCM2_chick]:
        mv.STATUS = 1

    # Smooth manure trajectories a bit (tune)
    for mv in [FCM_cow, FCM_pig, FCM_chick, FCM2_cow, FCM2_pig, FCM2_chick]:
        mv.DCOST = 1e-4

    # If you want no movement penalty for other MVs:
    for mv in [FDR, FDH, MP, MNO3, MTAN, ADR]:
        mv.DCOST = 0.0
    # -----------------------------
    # 6) Manure nutrient streams
    # -----------------------------
    Pman1   = m.Intermediate(FCM_cow*manure['cow']['P']   + FCM_pig*manure['pig']['P']   + FCM_chick*manure['chick']['P'])
    TANman1 = m.Intermediate(FCM_cow*manure['cow']['TAN'] + FCM_pig*manure['pig']['TAN'] +
FCM_chick*manure['chick']['TAN'])
    Norg1   = m.Intermediate(FCM_cow*manure['cow']['Norg'] + FCM_pig*manure['pig']['Norg'] +
FCM_chick*manure['chick']['Norg'])
    VS1     = m.Intermediate(FCM_cow*manure['cow']['VS']  + FCM_pig*manure['pig']['VS']  + FCM_chick*manure['chick']['VS'])

    Pman2   = m.Intermediate(FCM2_cow*manure['cow']['P']   + FCM2_pig*manure['pig']['P']   + FCM2_chick*manure['chick']['P'])
    TANman2 = m.Intermediate(FCM2_cow*manure['cow']['TAN'] + FCM2_pig*manure['pig']['TAN'] +
FCM2_chick*manure['chick']['TAN'])
    Norg2   = m.Intermediate(FCM2_cow*manure['cow']['Norg'] + FCM2_pig*manure['pig']['Norg'] +
FCM2_chick*manure['chick']['Norg'])
    VS2     = m.Intermediate(FCM2_cow*manure['cow']['VS']  + FCM2_pig*manure['pig']['VS']  +
FCM2_chick*manure['chick']['VS'])


    # -----------------------------
    # 7) States
    # -----------------------------
    CPR1  = m.SV(value=x0[0])
    CPR2  = m.SV(value=x0[1])
    CNO3R = m.SV(value=x0[2])
    CTANR = m.SV(value=x0[3])
    CNR   = m.SV(value=x0[4])
```

```
CPA   = m.SV(value=x0[5])
CTANA = m.SV(value=x0[6])

CPH   = m.SV(value=x0[8])
CNO3H = m.SV(value=x0[9])
CTANH = m.SV(value=x0[10])

CPP   = m.Var(value=150*sf)
CPTAN = m.Var(value=600*sf)

CODR1 = m.SV(value=0)
CODR2 = m.SV(value=0)
VSsum = m.Var(value=0)
# -----------------------------
# 8) Design / hydraulics
# -----------------------------
max_F = float(np.max(Fs))
FSW_max = max_F * c['DM'] * c['TSS'] * ((1 - c['sTSS']) / c['sTSS'])
F_AD1_max = FSW_max + FCM_max
FoutAD = F_AD1_max

Vad = F_AD1_max * c['HRT']  # L

# HPS demand
FEV = m.Intermediate(AHPS * c['Ac'] * ET)     # L/day
FSW = m.Intermediate(F * c['DM'] * c['TSS'] * ((1 - c['sTSS']) / c['sTSS']))

# AD1 outflow to HPS + discharge, etc.
FAD1 = m.Intermediate(FoutAD - FSW - FCM1_tot)   # L/day (your original)

# AD2 sizing
PW_VSmax = (np.mean(Es) * AHPS * c['Ac'] * (105 + 250) * 0.5 * 1e-3 *
            c['Pwaste'] * (1 / c['drm_N']) * (1 + ((1 - c['DMplant']) / c['DMplant'])) * c['VS'])
VS_avg = np.mean([c['PigVS'], c['ChickVS'], c['CowVS']])
CM_VSmax = FCM2_max * VS_avg * 0.3
Vpw = (PW_VSmax + CM_VSmax) / c['VOLR']  # L

# inflows
FHin = m.Intermediate(
    FEV + FDH*1000 - ADR*FDR*1000 - (FoutAD + (Vpw / c['HRT_pw'])) * 0.9
)
```

```
FRin = m.Intermediate(FSW + FDR*1000)

# -----------------------------
# 9) Core dynamics
# -----------------------------
m.Equation(CPR1.dt()  == (F*1e3*c['wP']*c['dF']*c['ePS'] - (FSW+FDR*1000)*CPR1) / c['Vras'])
m.Equation(CPR2.dt()  == (F*1e3*c['wP']*((1-c['dF'])+c['dF']*c['ePF']) - (FSW)*CPR2) / c['Vras'])
m.Equation(CNO3R.dt() == (F*1e3*c['wN']*c['dF']*c['eTAN']*c['bf']*(1-c['denRAS']) - (FSW+FDR*1000)*CNO3R) / c['Vras'])
m.Equation(CTANR.dt() == (F*1e3*c['wN']*c['dF']*c['eTAN']*(1-c['bf']) - (FSW+FDR*1000)*CTANR) / c['Vras'])
m.Equation(CNR.dt()   == (F*1e3*c['wN']*((1-c['dF'])+c['dF']*c['eNF']) - (FSW)*CNR) / c['Vras'])
m.Equation(CODR1.dt() == (F*1e3*c['wCOD']*c['dF']*c['eCOD'] - (FSW+FDR*1000)*CODR1) / c['Vras'])
m.Equation(CODR2.dt() == (F*1e3*c['wCOD']*((1-c['dF'])+c['dF']*c['eCODF']) - FSW*CODR2) / c['Vras'])

# AD1 concentrations
m.Equation(CPA.dt()   == (Pman1*c['iP'] + FSW*(CPR1+CPR2*c['iP']) - FoutAD*CPA) / Vad)
m.Equation(CTANA.dt() == (Norg1*c['iN'] + TANman1 + FSW*(CTANR + CNR*c['iN']) - FoutAD*CTANA) / Vad)

# AD2 concentrations
m.Equation(CPP.dt()   == (CPH*FEV*c['Pwaste']*c['pw_iP'] + Pman2*c['iP'] - CPP*(Vpw/c['HRT_pw'])) / Vpw)
m.Equation(CPTAN.dt() == (((CTANH+CNO3H)*FEV*c['Pwaste']*c['pw_iN']) + TANman2 + Norg2*c['iN'] -
CPTAN*(Vpw/c['HRT_pw'])) / Vpw)

# HPS dynamics
m.Equation(CPH.dt() == (
    FDR*ADR*1000*CPR1
    + 0.9*(FoutAD*CPA + (Vpw/c['HRT_pw'])*CPP)
    + MP*1e3
    - CPH*(FEV + FDH*1000)
) / (AHPS*0.9*0.2*1000))

m.Equation(CTANH.dt() == (
    FDR*ADR*1000*CTANR
    + 0.9*FoutAD*(CTANA*(1-BF) + CTANA*BF*0.05)
    + 0.9*(Vpw/c['HRT_pw'])*(CPTAN*(1-BF) + CPTAN*BF*0.05)
    + MTAN*1e3
    - CTANH*(FEV + FDH*1000)
) / (AHPS*0.9*0.2*1000))

m.Equation(CNO3H.dt() == (
    (FDR*ADR*1000*CNO3R
     + 0.9*FoutAD*CTANA*BF*0.95
```

```
        + 0.9*(Vpw/c['HRT_pw'])*CPTAN*BF*0.95
        + MNO3*1e3) * (1-c['denHPS'])
       - CNO3H*(FEV + FDH*1000)
    ) / (AHPS*0.9*0.2*1000))

    CNH = m.Intermediate(CTANH + CNO3H)

    # VS loading in AD2
    VSin = m.Intermediate(((FEV*(CNO3H+CTANH)*c['Pwaste']*(1/c['drm_N'])*(1 + ((1-c['DMplant'])/c['DMplant'])))*c['VS'] + VS2)
/ Vpw)
    m.Equation(VSsum.dt() == VSin)

    # running average VS (optional; keep your intent)
    time_horizon = float(ts[-1] - ts[0])
    VSavg = m.Var(value=float(VSin.value[0]) if hasattr(VSin, "value") else 0.0)
    m.Equation(VSavg * time_horizon == VSsum)

    # plant-waste flow and AD2 discharge term
    Fplant = m.Intermediate(((CTANH+CNO3H)*c['Pwaste']*FEV*(1/c['drm_N'])*1e-3*((1-c['DMplant'])/c['DMplant'])))
    FAD2 = m.Intermediate((Vpw/c['HRT_pw']) - FCM2_tot - Fplant)

    # -----------------------------
    s_flow     = m.Var(value=0.0, lb=0)
    s_FAD2     = m.Var(value=0.0, lb=0)
    s_FHin     = m.Var(value=0.0, lb=0)
    s_CTANR_hi = m.Var(value=0.0, lb=0)

    LB_N = float(LB[0]) * sf
    UB_N = float(UB[0]) * sf
    LB_P = float(LB[1]) * sf
    UB_P = float(UB[1]) * sf

    m.Equations([
       CNO3R >= 0,
       CPR1  >= 0,
       CPR2  >= 0,
       CTANR >= 0,
       CTANH >= 0,

       CTANH - 0.5*CNH <= 0,
```

```
    CNO3R - 200*sf <= 0,
    CTANR - 4*sf  <= 0,
    CTANR - 2*sf - s_CTANR_hi <= 0,

    CNH - UB_N <= 0,
    LB_N - CNH <= 0,

    CPH - UB_P <= 0,
    LB_P - CPH <= 0,
])
# slackened feasibility constraints
m.Equation(FAD2 + s_FAD2 >= 0)
m.Equation(FAD2 + FCM2_tot - (Vpw/c['HRT_pw']) <= s_flow)
m.Equation(FHin + s_FHin >= 0)
# -----------------------------
eps = 1e-6
CPH0   = x0[8]
CNH0   = x0[9] + x0[10]
CNO3R0 = x0[2]

track_HPS = ((CNH - CNH0)/(CNH0 + eps))**2 + ((CPH - CPH0)/(CPH0 + eps))**2
track_RAS = ((CNO3R - CNO3R0)/(CNO3R0 + eps))**2

VS_opt = 1.5
track_VS = ((VSin - VS_opt)/(VS_opt + eps))**2

manure_level = (FCM1_tot/FCM_max)**2 + (FCM2_tot/FCM2_max)**2

# weights (tune)
w_flow   = 1e3
w_track  = 1e1
w_vs     = 5.0
w_manure = 0.0
w_slack  = 1e3

L = m.Intermediate(
    w_flow  * (MP + MNO3 + MTAN + FDH + (1-ADR)*FDR)
    + w_track * (track_HPS + track_RAS)
    + w_vs    * track_VS
    + w_manure * manure_level
    + w_slack * (s_FHin + s_flow + s_FAD2 + s_CTANR_hi)
```

```
)

J = m.Var(value=0.0)
m.Equation(J.dt() == L)

# terminal-only objective: minimize J(tf)
wT_vec = np.zeros(len(ts))
wT_vec[-1] = 1.0
wT = m.Param(value=wT_vec)
m.Minimize(wT * J)
# -----------------------------
m.options.IMODE = 6
m.options.NODES = 3
m.options.SOLVER = 3
m.options.MAX_ITER = 1000
m.options.RTOL = 1e-5
m.options.OTOL = 1e-5

try:
    m.solve(disp=disp)
except Exception as e:
    print("Solve failed:", e)
    try:
        from gekko.apm import get_file
        f = get_file(m._server, m._model_name, 'infeasibilities.txt')
        f = f.decode().replace('\r', '')
        with open('infeasibilities.txt', 'w') as fl:
            fl.write(str(f))
        print("Wrote infeasibilities.txt")
    except Exception as e2:
        print("Could not fetch infeasibilities.txt:", e2)

df = build_timeseries_dataframe(
    m, c, F, ET,
    CPR1, CPR2, CNO3R, CTANR, CNR,
    CODR1, CODR2,
    CPA, CTANA, CPP, CPTAN,
    CPH, CTANH, CNO3H,

    FDR, FDH, FHin, FRin, FSW, FEV, FAD1, FAD2, FoutAD, Vpw/c['HRT_pw'], Fplant,
    FCM_cow, FCM_pig, FCM_chick, FCM2_cow, FCM2_pig, FCM2_chick,
```

```
    FCM1_tot, FCM2_tot, Pman1, Pman2, TANman1, TANman2, Norg1, Norg2,
    MP, MNO3, MTAN, ADR, BF,
    VSavg
)

KPIs = compute_kpis(df, c)

ctrl_traj = {
    'FDR': np.array(FDR.value) * 1000.0,
    'FDH': np.array(FDH.value) * 1000.0,

    'FCM1_tot': np.array(FCM1_tot.value),
    'FCM1_cow': np.array(FCM_cow.value),
    'FCM1_pig': np.array(FCM_pig.value),
    'FCM1_chick': np.array(FCM_chick.value),

    'FCM2_tot': np.array(FCM2_tot.value),
    'FCM2_cow': np.array(FCM2_cow.value),
    'FCM2_pig': np.array(FCM2_pig.value),
    'FCM2_chick': np.array(FCM2_chick.value),

    'MP': np.array(MP.value),
    'MNO3': np.array(MNO3.value),
    'MTAN': np.array(MTAN.value),
    'ADR': np.array(ADR.value),
    'BF': np.array(BF.value),

    'F': np.array(F.value),
    'ET': np.array(ET.value),
    'time': np.array(m.time),

    'slack_flow': np.array(s_flow.value),
    'slack_FAD2': np.array(s_FAD2.value),
    'slack_FHin': np.array(s_FHin.value),
    'slack_CTANR_hi': np.array(s_CTANR_hi.value),

    'J': np.array(J.value),
}

return df, KPIs, ctrl_traj
```

Supplemental references

S[1] Goddek, S., and Körner, O. (2019). A fully integrated simulation model of multi-loop aquaponics: a case study for system sizing in different environments. Agricultural systems *171*, 143-154.

S[2] Jansen, L., and Keesman, K.J. (2022). Exploration of efficient water, energy and nutrient use in aquaponics systems in northern latitudes. Cleaner and Circular Bioeconomy *2*, 100012.

S[3] Dijkgraaf, K.H., Goddek, S., and Keesman, K.J. (2019). Modeling innovative aquaponics farming in Kenya. Aquaculture International *27*, 1395-1422.

S[4] Montanhini Neto, R., and Ostrensky, A. (2015). Nutrient load estimation in the waste of Nile tilapia Oreochromis niloticus (L.) reared in cages in tropical climate conditions. Aquaculture Research *46*, 1309-1322.

S[5] Mirzoyan, N., Parnes, S., Singer, A., Tal, Y., Sowers, K., and Gross, A. (2008). Quality of brackish aquaculture sludge and its suitability for anaerobic digestion and methane production in an upflow anaerobic sludge blanket (UASB) reactor. Aquaculture *279*, 35-41.

S[6] Mirzoyan, N., Tal, Y., and Gross, A. (2010). Anaerobic digestion of sludge from intensive recirculating aquaculture systems. Aquaculture *306*, 1-6.

S[7] Mirzoyan, N., McDonald, R.C., and Gross, A. (2012). Anaerobic treatment of brackishwater aquaculture sludge: an alternative to waste stabilization ponds. Journal of the World Aquaculture Society *43*, 238-248.

S[8] Delaide, B., Goddek, S., Gott, J., Soyeurt, H., and Jijakli, M.H. (2016). Lettuce (Lactuca sativa L. var. Sucrine) growth performance in complemented aquaponic solution outperforms hydroponics. Water *8*, 467.

S[9] Ebeling, J.M., and Timmons, M.B. (2010). Recirculating aquaculture (Cayuga Aqua Ventures Ithaca, NY, USA).

S[10] Zhu, Z., Yogev, U., Keesman, K.J., Rachmilevitch, S., and Gross, A. (2023). Integrated hydroponics systems with anaerobic supernatant and aquaculture effluent in desert regions: Nutrient recovery and benefit analysis. Science of the Total Environment *904*, 166867.

S[11] Jung, I., and Lovitt, R. (2011). Leaching techniques to remove metals and potentially hazardous nutrients from trout farm sludge. Water research *45*, 5977-5986.

S[12] Zhu, Z., Yogev, U., Keesman, K.J., and Gross, A. (2021). Onsite anaerobic treatment of aquaponics lettuce waste: digestion efficiency and nutrient recovery. Aquaculture International *29*, 57-73.

S[13] Goddek, S., and Vermeulen, T. (2018). Comparison of Lactuca sativa growth performance in conventional and RAS-based hydroponic systems. Aquaculture International *26*, 1377-1386.

S[14] Mirzoyan, N., and Gross, A. (2013). Use of UASB reactors for brackish aquaculture sludge digestion under different conditions. Water research *47*, 2843-2850.

S[15] Van Lier, J.B., Mahmoud, N., and Zeeman, G. (2008). Anaerobic wastewater treatment. Biological wastewater treatment: principles, modelling and design, 415-456.

S[16] de Korte, M., Bergman, J., van Willigenburg, L.G., and Keesman, K.J. (2024). Towards a zero-waste aquaponics-centered eco-industrial food park. Journal of Cleaner Production *454*, 142109.

S[17] Gebauer, R., and Eikebrokk, B. (2006). Mesophilic anaerobic treatment of sludge from salmon smolt hatching. Bioresource technology *97*, 2389-2401.

S[18] Srivichai, P., and Chavalparit, O. (2020). Co-digestion of modified tapioca starch sludge and shrimp pond sediment as a method to improve system stability and biogas production. ScienceAsia *46*, 119-127.

S[19] Babæe, A., and Shayegan, J. (2011). Effect of organic loading rates (OLR) on production of methane from anaerobic digestion of vegetables waste. pp. 8-13.

S[20] CBAV (2025). Composition of different types of livestock manure. https://www.handboekbodemenbemesting.nl/bemestingsadviezen/samenstelling-organische-meststoffen/.

S[21] Liu, Z.-G., Zhou, X.-F., Zhang, Y.-L., and Zhu, H.-G. (2012). Enhanced anaerobic treatment of CSTR-digested effluent from chicken manure: the effect of ammonia inhibition. Waste management *32*, 137-143.

S[22] Zippenfenig, P. (2023). Open-Meteo.com Weather API. Zenodo. .

S[23] Tchobanoglous, G., Stensel, H.D., Tsuchihashi, R., Burton, F., Abu-Orf, M., Bowden, G., and Pfrang, W. (2014). Wastewater engineering: treatment and resources recovery. Metcalf and Eddy Inc.

S[24] Cheng, H., Li, Y., Hu, Y., Guo, G., Cong, M., Xiao, B., and Li, Y.-Y. (2021). Bioenergy recovery from methanogenic co-digestion of food waste and sewage sludge by a high-solid anaerobic membrane bioreactor (AnMBR): mass balance and energy potential. Bioresource Technology *326*, 124754.

S[25] Puchajda, B., and Oleszkiewicz, J. (2008). Impact of sludge thickening on energy recovery from anaerobic digestion. Water Science and Technology *57*, 395-401.
